# The insulin receptor family and protein kinase B (Akt) are activated in the heart by alkaline pH and α_1_-adrenergic receptors

**DOI:** 10.1042/BCJ20210144

**Published:** 2021-06-08

**Authors:** Daniel N. Meijles, Stephen J. Fuller, Joshua J. Cull, Hajed O. Alharbi, Susanna T.E. Cooper, Peter H. Sugden, Angela Clerk

**Affiliations:** 1 School of Biological Sciences, University of Reading, Reading RG6 6AS, U.K.; 2 Molecular and Clinical Sciences Institute, St George's University of London, London SW17 0RE, U.K.

**Keywords:** Gq protein coupled receptors, protein synthesis, receptor cross-talk

## Abstract

Insulin and insulin-like growth factor stimulate protein synthesis and cardioprotection in the heart, acting through their receptors (INSRs, IGF1Rs) and signalling via protein kinase B (PKB, also known as Akt). Protein synthesis is increased in hearts perfused at alkaline pH_o_ to the same extent as with insulin. Moreover, α_1_-adrenergic receptor (α_1_-AR) agonists (e.g. phenylephrine) increase protein synthesis in cardiomyocytes, activating PKB/Akt. In both cases, the mechanisms are not understood. Our aim was to determine if insulin receptor-related receptors (INSRRs, activated in kidney by alkaline pH) may account for the effects of alkaline pH_o_ on cardiac protein synthesis, and establish if α_1_-ARs signal through the insulin receptor family. Alkaline pH_o_ activated PKB/Akt signalling to the same degree as insulin in perfused adult male rat hearts. INSRRs were expressed in rat hearts and, by immunoblotting for phosphorylation (activation) of INSRRs/INSRs/IGF1Rs, we established that INSRRs, together with INSRs/IGF1Rs, are activated by alkaline pH_o_. The INSRR/INSR/IGF1R kinase inhibitor, linsitinib, prevented PKB/Akt activation by alkaline pH_o_, indicating that INSRRs/INSRs/IGF1Rs are required. Activation of PKB/Akt in cardiomyocytes by α_1_-AR agonists was also inhibited by linsitinib. Furthermore, linsitinib inhibited cardiomyocyte hypertrophy induced by α_1_-ARs in cultured cells, reduced the initial cardiac adaptation (24 h) to phenylephrine *in vivo* (assessed by echocardiography) and increased cardiac fibrosis over 4 days. We conclude that INSRRs are expressed in the heart and, together with INSRs/IGF1Rs, the insulin receptor family provide a potent system for promoting protein synthesis and cardioprotection. Moreover, this system is required for adaptive hypertrophy induced by α_1_-ARs.

## Introduction

Cardiac muscle contains terminally differentiated cardiomyocytes for contraction, a dense network of capillaries for efficient delivery of oxygen and nutrients, and resident fibroblasts providing extracellular matrix to maintain a robust structure. Although cardiomyocytes do not divide, they can hypertrophy (i.e. increase their size and myofibrillar content), allowing the heart to accommodate an increase in workload [1,2]. For example, in pregnancy or with sustained/repeated exercise, this ‘physiological' hypertrophy is not associated with increased inflammation or fibrosis, and is reversible. However, sustained stress on the heart (e.g. hypertension) may cause adverse remodelling with cardiomyocyte damage, inflammation, loss of capillaries and enhanced fibrosis. Here, the initial adaptive or ‘compensated' hypertrophy is not sustained, remodelling becomes irreversible, and ‘decompensated' hypertrophy develops, leading towards heart failure.

Cardiomyocyte hypertrophy is associated with changes in gene expression that facilitate cell growth [1,3]. This includes expression of immediate early genes and a switch to a ‘foetal' gene programme with re-expression of, for example, *Myh7* encoding β myosin heavy chain, *Nppa* encoding atrial natriuretic factor and *Nppb* encoding B-type natriuretic factor. As cardiomyocytes increase in size, cardiac remodelling is required, with modification of the extracellular matrix and an increase in the capillary network [1,2]. Cardiomyocyte hypertrophy is associated with increased protein synthesis to facilitate cell growth, and cytoprotective systems are required to prevent cell death. Two key signalling pathways drive many of these changes: (i) PKB/Akt signalling is cytoprotective and increases protein synthesis [4,5]; (ii) the extracellular signal-regulated kinases 1/2 (ERK1/2) also promote translation and confer cytoprotection [5], but are particularly important in effecting changes in gene expression during cardiomyocyte hypertrophy [3,6,7]. Gq protein-coupled receptors (GqPCRs) such as α_1_-adrenergic receptors (α_1_-ARs) promote cardiomyocyte hypertrophy, and α_1_-AR agonists (e.g. phenylephrine or the selective α_1A_-AR agonist A61603) potentially prevent cardiac maladaptation [8]. These agonists activate both ERK1/2 and PKB/Akt in cardiomyocytes [9].

The ERK1/2 pathway is activated by receptor tyrosine kinases and GqPCRs, and has been extensively studied in the context of cardiomyocyte hypertrophy [3]. Here, the small G protein Ras activates Raf kinases that phosphorylate/activate mitogen-activated protein kinase (MAPK) kinases 1/2 (MKK1/2) which phosphorylate/activate ERK1/2. ERK1/2 phosphorylate downstream substrates including p90 ribosomal S6 kinases (p90RSKs) to increase protein synthesis, promote cardioprotection and elicit changes in gene expression in cardiomyocytes [7,10]. ERK1/2 are the prototypic MAPKs. Other MAPKs such as p38-MAPK and c-Jun N-terminal kinases (JNKs) are generally stress-responsive and associated with maladaptive hypertrophy [11].

The PKB/Akt pathway was first defined in response to insulin [12]. The insulin receptor (INSR) is a heterotetramer of two extracellular α subunits linked to two transmembrane β subunits via disulfide bonds, with the tyrosine kinase located in the intracellular domain of the β subunits [13]. Insulin binding to insulin receptors promotes Tyr phosphorylation of the intracellular β subunits, recruiting phosphoinositide 3′ kinase (PI3K) to the receptor complex and resulting in phosphorylation and activation of PKB/Akt [12]. Downstream substrates become phosphorylated including glycogen synthase kinase 3α and β (GSK3α/β, which are inhibited), and p70 ribosomal S6 kinases (p70S6K) which phosphorylate the small ribosomal subunit Rps6 to regulate translation of specific mRNAs, particularly increasing the protein synthetic machinery. Additionally, PKB/Akt-dependent signalling via mammalian target of rapamycin (mTOR) influences the rate of protein synthesis. Insulin-like growth factor 1 (IGF1) binds to a similar receptor (IGF1R), activates the same pathways, and is particularly associated with physiological hypertrophy [14]. A third member of the insulin receptor family, the insulin receptor-related receptor (INSRR) was identified in 1989, having a similar overall structure to INSR, with a divergent α subunit and high homology with the INSR kinase domain [15]. INSRRs have no established ligand, but are activated by alkali, responding to increased extracellular pH_o_ [16]. Notably, perfusion of rat hearts at alkaline pH_o_ promotes protein synthesis to a similar degree as insulin [17].

 In this study, we established that INSRRs are expressed and functional in the heart. We also identified a novel signalling paradigm in cardiomyocytes in which α_1_-ARs transactivate insulin receptor family members leading to activation of PKB/Akt and ERK1/2. Inhibition of insulin receptor family members with linsitinib (OSI-906), a drug developed for cancer [18], compromised this pathway, inhibiting cardiac adaptation in phenylephrine-induced hypertrophy *in vivo*, causing increased fibrosis. Thus, insulin receptor family signalling is required for physiological hypertrophy induced by α_1_-ARs.

## Materials and methods

### Ethics statement for animal experiments

Male C57Bl/6J mice (7 weeks), adult male Sprague–Dawley rats (300–350 g) and female Sprague–Dawley rats with 2–4 day neonates were purchased from Charles River (U.K.) and imported into the BioResource Unit at University of Reading (with a U.K. Home Office certificate of designation). Studies were performed in accordance with European Parliament Directive 2010/63/EU on the protection of animals used for scientific purposes, local institutional animal care committee procedures (University of Reading) and the U.K. Animals (Scientific Procedures) Act 1986 (Procedure Project Licences 70/8248, 70/8249, and P8BAB0744).

### Animal housing, husbandry and welfare

Mice were housed in Tecniplast IVC cages (total area 512 cm^2^; maximum five mice per cage). Adult male rats were housed in open top NKP cages (total area 1632 cm^2^; maximum five rats per cage). Cages were supplied with aspen sawdust bedding, sizzle nesting, cardboard tunnels and housing. Additional enrichment included chew sticks and millet to encourage foraging behaviour. Animals were provided with water and food (SDS Rm3 pelleted food for mice; SDS RM3 expanded pelleted food for rats) *ad libitum*, with a 12 : 12 light/dark cycle and room temperature of 21°C. All animals were checked at least once a day by a trained, competent person and licence holders informed of any welfare issues, with consultation with a Named Veterinary Surgeon when necessary. Mice undergoing procedures were monitored using a score sheet and routinely culled if they reached a predefined endpoint agreed with the Named Veterinary Surgeon. Weights were taken before, during and at the end of the procedures. Mouse weights from the start and end of procedures are provided in Supplementary Table S1. Mice were excluded after randomisation only if there was a health problem unrelated to the study. All animals were fully randomised and assigned to study groups before experimentation. *In vivo* experiments with mice commenced when animals were 10–12 weeks of age.

### *In vivo* mouse studies

Drug delivery used Alzet osmotic pumps (models 1007D or 1002; supplied by Charles River U.K.), filled according to the manufacturer's instructions in a laminar flow hood using sterile technique. Mice were treated with phenylephrine (40 mg/kg/d) dissolved in PBS, with DMSO/PEG mix [50% (v/v) dimethyl sulphoxide (DMSO), 20% (v/v) polyethylene glycol 400, 5% (v/v) propylene glycol, 0.5% (v/v) Tween 80] or linsitinib (2.0 mg/kg/d) dissolved in DMSO/PEG mix. Linsitinib was from Selleck Chemicals; phenylephrine and vehicle components were from Sigma–Aldrich. Minipumps were incubated overnight in sterile PBS (37°C) then implanted in the mice under continuous inhalation anaesthesia using isoflurane (induction at 5%, maintenance at 2–2.5%) mixed with 2 l/min O_2_. A 1 cm incision was made in the mid-scapular region and mice were given 0.05 mg/kg (s.c.) buprenorphine (Ceva Animal Health Ltd.) to repress post-surgical discomfort. Minipumps were implanted portal first in a pocket created in the left flank region of the mouse. Wound closure used a simple interrupted suture with polypropylene 4-0 thread or two wound clips. Mice were recovered singly and returned to their home cage once fully recovered.

Echocardiography was performed on anaesthetised mice using a Vevo 2100 imaging system with a MS400 18–38 MHz transducer (Visualsonics). Mice were anaesthetised in an induction chamber with isoflurane (5% flow rate) with 1 l/min O_2_ then transferred to the heated Vevo Imaging Station. Anaesthesia was maintained with 1.5% isoflurane delivered via a nose cone. Left ventricular cardiac function and structure was assessed from short axis B-mode (for interventricular septum) or M-mode (all other measurements) images with the axis placed at the mid-level of the left ventricle at the level of the papillary muscles. Baseline scans were taken prior to experimentation (−7 to −3 days). Further scans were taken at 24 h and 4 days post-minipump implantation. Imaging was completed within 20 min. Mice were recovered singly and transferred to the home cage once fully recovered. Data analysis used Vevo Lab software (Visualsonics) and was performed by independent assessors blinded to intervention. Data were gathered from two scans taken from each time point, taking mean values across at least three cardiac cycles for each scan. Images were exported from the Vevo Lab software and cropped for presentation using Adobe Photoshop CC, maintaining the relative proportions of the echocardiograms.

Mice were euthanised by CO_2_ inhalation followed by cervical dislocation. Hearts were excised quickly, washed in PBS and snap-frozen in liquid N_2_ or fixed for histology.

### Histology and assessment of myocyte size and fibrosis

Histological analysis was performed on hearts fixed with 10% formalin. Hearts were immersed in 70% (v/v) ethanol, embedded in paraffin and sectioned at 10 µm. Sections were de-waxed using xylene and re-hydrated through sequential washes in decreasing ethanol gradients (100%, 100%, 90%, 75%, 50%) to distilled water. Sections were stained using kits for haematoxylin and eosin (H&E, Sigma) or Masson's trichrome (Polysciences). For H&E staining, sections were submerged in Harris haematoxylin, differentiated in 1% acid alcohol, ‘blued’ in saturated lithium carbonate, and counterstained in eosin Y solution. For Masson's trichrome staining, sections were incubated in Bouin's fixative (60 min, 60°C) then processed through Weigert's iron hematoxylin, Biebrich Scarlet-acid fuchsin, and aniline blue with 1% acetic acid differentiation stains. Sections were rapidly dehydrated to xylene and mounted in DPX for image capture using a Hamamatsu slide scanner.

For analysis of myocyte cross-sectional area, cells within the left ventricle (excluding endocardial regions) were chosen at random and outline traced using NDP.view2 software (Hamamatsu). Only cells with a single nucleus that were clearly in cross-section were included in the analysis. For assessment of fibrosis, 20× images of the entire left ventricle were exported and the collagen fraction calculated as the ratio between the sum of the total area of fibrosis (blue colour) to the sum of the total tissue area (including the myocyte area) for the entire image using ImageJ. All histological and data analysis was performed by independent assessors blinded to treatment groups.

### Adult rat heart perfusions

Adult male (300–350 g) Sprague–Dawley rats (Charles River) were anaesthetised with a lethal intraperitoneal dose of pentobarbital sodium (60 mg/kg). After complete anaesthesia was induced, heparin (1000 U/kg) was administered intravenously. The chest cavity was opened and the heart and lungs were removed into modified (high KCl) ice-cold Krebs–Henseleit bicarbonate-buffered saline (KHBBS: 25 mM NaHCO_3_, 119 mM NaCl, 35 mM KCl, 2.5 mM CaCl_2_, 1.2 mM MgSO_4_, 1.2 mM KH_2_PO_4_ equilibrated with 95% O_2_/5% CO_2_) whilst the heart was still beating. Surrounding tissues were removed from the heart before aortic cannulation and perfusion.

Three perfusion buffers were used (i) KHBBS (25 mM NaHCO_3_, 119 mM NaCl, 4.7 mM KCl, 2.5 mM CaCl_2_, 1.2 mM MgSO_4_, 1.2 mM KH_2_PO_4_, pH 7.4, containing 10 mM glucose and equilibrated with 95% O_2_/5% CO_2_); (ii) nominally CO_2_/HCO_3_^–^-free modified Tris-buffered Tyrode's solution (10 mM Tris base, 140 mM NaCl, 6 mM KCl, 1 mM MgCl_2_, 2 mM CaCl_2_ containing 10 mM glucose, with the pH adjusted to 7.4 at 21°C with 12 M HCl) equilibrated with 100% O_2_, or (iii) modified Tyrode's solution as in (ii) with the pH adjusted to 9.1 at 21°C with 12 M HCl. The pH of the pH 9.1 buffer falls during recirculating perfusions presumably because of absorption of CO_2_ and lactic acid release by the heart [19]. Thus, the pH values of the Tris-containing buffers are nominal and denoted by pH ‘7.4’ or ‘9.1’.

Hearts were perfused retrogradely at constant pressure (70 mm Hg) with pre-perfusion with KHBBS (15 min, 37°C). When the perfusate in the experimental period differed from that in the pre-perfusion period, it was switched after the pre-perfusion period using a second inlet line. Agonists or inhibitors were present in the perfusates at the following concentrations: 50 mU/ml insulin (Novo-Nordisk), 50 nM A61603 (Tocris Bioscience), and/or 50 µM LY294002 or linsitinib (Selleck Chemicals). In some cases, global ischaemia (20 min) with reperfusion (40 min) was used as a positive control for p38-MAPK or JNK phosphorylation [20]. In this case, ischaemia was imposed by closing the aortic perfusion line. For immunoblotting, at the end of the perfusions, hearts were ‘freeze-clamped’ between aluminium tongs cooled in liquid N_2_ and were promptly pulverised under liquid N_2_ in a pestle and mortar. The resulting powders were stored at −80°C.

The effect of pH on the rate of protein synthesis in perfused rat hearts was measured by incorporation of [U-^14^C]-L-phenylalanine (PerkinElmer) in the presence of all the amino acids required for protein synthesis. Hearts were pre-perfused (5 min) with Tris-buffered Tyrode's or modified Tyrode's buffer as defined above. Hearts were then perfused for 2 h with buffers supplemented with amino acids (0.2 mM for all except phenylalanine, which was at 0.4 mM) containing 0.37 mBq [U-^14^C]-L-phenylalanine. Ventricles were collected, ‘freeze-clamped’ and pulverised under liquid N_2_ as above. The resulting powders were stored at −80°C. Weighed samples were dissolved in 0.2 M NaOH with incubation at 37°C for 4 h. Samples were taken for protein assay (Bio-Rad Bradford) using bovine serum albumin (BSA) standards. Proteins were precipitated with trichloroacetic acid [5% (w/v) final concentration] and pelleted by centrifugation (100×***g***, 6 min, 4°C). Pellets were washed in 5% (w/v) trichloroacetic acid and dissolved in 2 ml Soluene 350 (PerkinElmer) with warming to 37°C. Samples were mixed thoroughly with Ultima Gold scintillation fluid (PerkinElmer) and [U-^14^C]-L-phenylalanine measured by scintillation counting.

### Preparation of membrane fractions enriched in T tubules

Membrane fractions enriched in T tubules were prepared essentially as described by Tishkoff et al. [21]. Adult male Sprague–Dawley rat hearts were removed as described for heart perfusions (above). Hearts were flushed with ice-cold PBS, followed by TKED buffer [50 mM Tris–HCl pH 7.4, 150 mM KCl, 1.5 mM ethylenediamine tetraacetic acid (EDTA), 10 mM dithiothreitol (DTT), 10 mM benzamidine, 0.2 mM leupeptin, 0.01 mM trans-epoxy succinyl-l-leucylamido-(4-guanidino)butane, 0.3 mM phenylmethylsulphonyl fluoride, 4 µM microcystin]. Hearts were trimmed free of atria and pericardium, minced finely with scissors, washed/resuspended in TKED buffer and homogenised on ice with short bursts using a Polytron homogeniser. Extracts were centrifuged (8600×***g***, 15 min, 4°C). Supernatants were removed and recentrifuged (47 800×***g***, 15 min, 4°C). Pellets from each centrifugation were resuspended in TKED buffer. Samples of whole heart extracts and resuspended membrane pellets were taken for protein assay. Protein concentrations were determined by Bio-Rad Bradford assay using BSA standards. Extracts were boiled with 0.33 vol sample buffer (300 mM Tris–HCl pH 6.8, 10% (w/v) SDS, 13% (v/v) glycerol, 130 mM dithiothreitol, 0.2% (w/v) bromophenol blue) for immunoblotting.

### Neonatal rat ventricular cardiomyocyte cultures

Neonatal rat cardiomyocytes were prepared and cultured from 3–4 day Sprague–Dawley rats (Charles River) essentially as previously described [22]. Neonatal rats were culled by cervical dislocation and then were decapitated. Ventricles were dissected and dissociated by serial digestion at 37°C with 0.44 mg/ml (6800 U) Worthington Type II collagenase (supplied by Lonza) and 0.6 mg/ml pancreatin (Sigma–Aldrich, cat. No. P3292) in sterile digestion buffer (116 mM NaCl, 20 mM HEPES, 0.8 mM Na_2_HPO_4_, 5.6 mM glucose, 5.4 mM KCl and 0.8 mM MgSO_4_, pH 7.35). The first digestion supernatant (5 min, 37°C, 160 cycles/min in a shaking waterbath) was discarded. Cell suspensions from subsequent digestions (4 × 25 min, 37°C 136 cycles/min shaking) were recovered by centrifugation (5 min, 60×***g***) and the cell pellet resuspended in plating medium (Dulbecco's modified Eagle's medium (DMEM)/medium 199 [4 : 1 (v/v)]) containing 15% (v/v) foetal calf serum (Life Technologies) and 100 U/ml penicillin and streptomycin. Cells were pre-plated on plastic tissue culture dishes (30 min) to remove non-cardiomyocytes. Non-adherent cardiomyocytes were collected and viable cells counted by Trypan Blue (Sigma–Aldrich) exclusion using a haemocytometer. For immunoblotting, viable cardiomyocytes were plated at a density of 4 × 10^6^ cells/dish on 60 mm Primaria dishes pre-coated with sterile 1% (w/v) gelatin (Sigma–Aldrich). After 18 h, myocytes were confluent and beating spontaneously. For immunostaining experiments, cardiomyocytes were plated at 1 × 10^6^ cells/dish on 35 mm Primaria dishes containing glass coverslips pre-coated with sterile 1% (w/v) gelatin followed by laminin (20 µg/ml in PBS; Sigma–Aldrich). For all experiments, the plating medium was withdrawn after 18 h and cells were incubated in serum-free maintenance medium (DMEM/medium 199 [4 : 1 (v/v)] containing 100 units/ml penicillin and streptomycin) for a further 24 h prior to experimentation.

### Generation of HEK293 cells expressing human INSRR

Sub-confluent HEK293 cells in 60 mm dishes were transformed using FuGENE® HD (Promega) with 10 µg of TrueORF Gold Expression-validated cDNA clone RC224956 (accession no. NM_014215) from Origene, which harbours a Myc-DDK (FLAG®)-tagged open reading frame clone of the INSRR in the pCMV-6 Entry Vector. Cells were then subjected to selection with 400 µg/ml G418 through multiple rounds of passaging until a constant level of expression of the transgene (monitored with an anti-FLAG® western blot) was observed ( ∼3 weeks). Cells were subsequently expanded, aliquoted and frozen for later use. Cells from frozen stocks were plated on 100 mm dishes in 4 ml of DMEM containing 10% foetal calf serum, 2 mM L-glutamine, 100 U/ml penicillin, 100 U/ml streptomycin and 400 µg/ml G418, and were incubated at 37°C in an atmosphere of 5% CO_2_ overnight. The cells were subsequently passaged into 60 mm dishes for the following day, by which time they were approaching confluence. The DMEM medium was subsequently replaced with 4 ml of Tyrode's solution containing 10 mM glucose adjusted to pH 7.4 or pH 9.1 with HCl, which had been warmed to 37°C and gassed extensively with O_2_. All incubations were for 1 min in an atmosphere of humidified air at 37°C. For INSRR phosphorylation, the cells were exposed to buffer at pH 7.4 or pH 9.1. For INSR or IGF1R phosphorylation, the cells were exposed to buffer at pH 7.4 containing insulin (50 mU/ml). When the effects of linsitinib (from Selleck Chemicals, prepared as 1000-fold-concentrated stocks in DMSO) were studied, cells were pre-incubated for 5 min in DMEM in the presence or absence of appropriate concentrations of linsitinib. DMSO (1/1000-diluted) was added to the (no linsitinib) controls. The medium was then changed and cells were exposed to buffers at pH 9.1 or 7.4 (defined above) containing insulin (50 mU/ml) and the appropriate concentrations of linsitinib.

### RNA preparation and qPCR

Total RNA was prepared using RNA Bee (AMS Biotechnology Ltd) using 1 ml per 10–15 mg mouse heart powder. RNA was prepared according to the manufacturer's instructions, dissolved in nuclease-free water and purity assessed from the A_260_/A_280_ measured using an Implen NanoPhotometer (values of 1.8–2.1 were considered acceptable). RNA concentrations were determined from the A_260_ values. Quantitative PCR (qPCR) analysis was performed as previously described [22]. Total RNA (1 µg) was reverse transcribed to cDNA using High Capacity cDNA Reverse Transcription Kits with random primers (Applied Biosystems) according to the manufacturer's instructions. qPCR was performed using an ABI Real-Time PCR 7500 system (Applied Biosystems) using 1/40 of the cDNA produced. Optical 96-well reaction plates were used with iTaq Universal SYBR Green Supermix (Bio-Rad Laboratories Inc.) according to the manufacturer's instructions. See Supplementary Table S2 for primer sequences. Results were normalised to *Gapdh*, and relative quantification was obtained using the ΔCt (threshold cycle) method; relative expression was calculated as 2^−ΔΔCt^, and normalised to vehicle.

### Immunoblotting

Hearts were ground to powder under liquid N_2_ and samples (15–20 mg) were extracted in 4 vol (rat hearts) or 6 vol (mouse hearts) Buffer A [20 mM β-glycerophosphate (pH 7.5), 50 mM NaF, 2 mM EDTA, 1% (v/v) Triton X-100, 5 mM dithiothreitol] containing protease and phosphatase inhibitors [10 mM benzamidine, 0.2 mM leupeptin, 0.01 mM trans-epoxy succinyl-l-leucylamido-(4-guanidino)butane, 0.3 mM phenylmethylsulphonyl fluoride, 4 µM microcystin]. For analysis of protein kinases in total cell extracts, cells were washed with ice-cold PBS and scraped into 150 µl Buffer A containing protease and phosphatase inhibitors. All samples were vortexed and extracted on ice (10 min), followed by centrifugation (10 000×***g***, 10 min, 4°C). The supernatants were removed, a sample was taken for protein assay and the rest boiled with 0.33 vol sample buffer (300 mM Tris–HCl pH 6.8, 10% (w/v) SDS, 13% (v/v) glycerol, 130 mM dithiothreitol, 0.2% (w/v) bromophenol blue). Protein concentrations were determined by Bio-Rad Bradford assay using BSA standards.

Proteins were separated by SDS–PAGE on 10% (w/v) polyacrylamide resolving gels with 6% stacking gels, and transferred electrophoretically to nitrocellulose using a Bio-Rad semi-dry transfer cell (10 V, 60 min). Non-specific binding sites were blocked with 5% (w/v) non-fat milk powder in Tris-buffered saline (20 mM Tris–HCl pH 7.5, 137 mM NaCl) containing 0.1% (v/v) Tween 20 (15 min). Blots were incubated with primary antibodies in Tris-buffered saline/Tween 20 containing 5% (w/v) BSA overnight at 4°C. The blots were washed with Tris-buffered saline/Tween 20 (3 × 5 min, 21°C), incubated with horseradish peroxidase-conjugated secondary antibodies (1 : 5000 dilution in Tris-buffered saline/Tween 20 containing 1% (w/v) non-fat milk powder, 60 min, 21°C) and then washed again in Tris-buffered saline/Tween 20 (3 × 5 min, 21°C). Details of antibodies used are in Supplementary Table S3. Bands were detected by enhanced chemiluminescence using ECL Prime Western Blotting detection reagents with visualisation using an ImageQuant LAS4000 system (GE Healthcare). ImageQuant TL 8.1 software (GE Healthcare) was used for densitometric analysis. Raw values for phosphorylated kinases were normalised to the total kinase. Values for all samples were normalised to the mean of the controls. Images were cropped for presentation using Adobe Photoshop CC.

### Immunostaining and planimetry

Neonatal cardiomyocytes were washed with ice-cold PBS and fixed in 3.7% (v/v) formaldehyde in PBS (10 min, room temperature). Cells were permeabilised with 0.3% (v/v) Triton X-100 (10 min, room temperature) in PBS, and non-specific binding blocked with 1% (w/v) fatty acid free BSA (Sigma–Aldrich U.K.) in PBS containing 0.3% (v/v) Triton X-100 (10 min, room temperature). All incubations were at room temperature in a humidified chamber, and coverslips were washed three times in PBS after each stage of the immunostaining procedure. Cardiomyocytes were stained with mouse monoclonal primary antibodies to troponin T (60 min) with detection using anti-mouse immunoglobulin secondary antibodies coupled to Alexa-Fluor 488 (60 min) (see Supplementary Table S3). Coverslips were mounted using fluorescence mounting medium (Dako) and viewed with a Zeiss Axioskop fluorescence microscope using a 40× objective. Digital images were captured using a Canon PowerShot G3 camera using a 1.8× digital zoom and cardiomyocyte sizes measured using ImageJ. Images were cropped for presentation using Adobe Photoshop CC.

### Statistical analysis

Data analysis used Microsoft Excel and GraphPad Prism 8.0. Statistical analysis was performed using GraphPad Prism with two-tailed *t*-tests, or two-tailed one-way or two-way ANOVA as indicated in the Figure legends. A multiple comparison test was used in combination with ANOVA as indicated in the Figure legends. A Grubb's outlier test was applied to the data, and outliers excluded from the analysis. Graphs were plotted with GraphPad Prism 8.0. Specific P values are provided with significance levels of *P* < 0.05 indicated.

## Results

### Increased alkalinity promotes cardiac protein synthesis via PI3K, activating PKB/Akt and MAPKs

**Figure 1.**
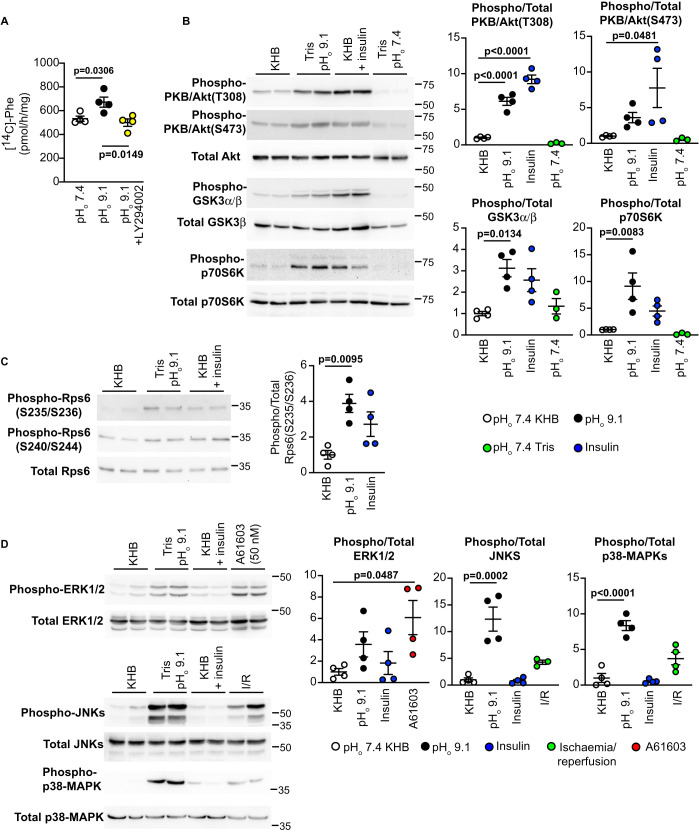
Alkaline pH signals to protein synthesis in perfused hearts via PI3K, activating PKB/Akt and MAPKs. Male rat hearts were perfused in the Langendorff mode (30 min) with Tris buffers at starting pH_o_ 7.4 or 9.1 with/without 50 µM LY294002, Krebs Henseleit buffer (KHB) alone or in the presence of 50 mU/ml insulin or 50 nM A61603, or were subjected to ischaemia (20 min) with reperfusion in KHB (40 min). (**A**) The rate of protein synthesis was measured by incorporation of [^14^C]-Phe. (**B**–**D**) Protein samples were immunoblotted with antibodies to phosphorylated (Phospho-) or total kinases or Rps6 as indicated. Representative blots are on the left (positions of relative molecular mass markers are indicated). Densitometric analysis (ratio of phosphorylated/total protein) is on the right of each set of immunoblots, with individual data points shown and means ± SEM (*n* = 3–4 per group). Statistical analysis used one-way ANOVA with Holm–Sidak post-test.

Insulin promotes protein synthesis via PI3K and PKB/Akt. As in previous studies [17], alkaline pH_o_ (pH_o_ 9.1; note, this is the initial buffer pH which falls to ∼pH 8.0 during the perfusion as CO_2_ is absorbed and lactate is released) increased protein synthesis in Langendorff perfused rat hearts (Figure 1A); this was significantly inhibited by the PI3K inhibitor, LY294002 (50 µM). Alkaline pH_o_ also activated PKB/Akt signalling to a comparable degree as insulin, with phosphorylation and activation of PKB/Akt itself, and phosphorylation of downstream pathway components (GSK3α/β, p70S6K, Rps6) (Figure 1B,C). ERK1/2 can increase the rate of protein synthesis in the heart [23], and were activated to some degree by alkaline pH_o_ (Figure 1D). ERK1/2 were also activated by the selective α_1A-_AR agonist, A61603, but insulin did not increase ERK1/2 activity to any significant extent, consistent with our previous studies in cardiomyocytes [24]. Exposure to pH_o_ 9.1 is a severe stress and, as expected, stress-regulated MAPKs (JNKs, p38-MAPKs) were activated to a high level, comparable with an alternative severe stressor, ischaemia with reperfusion (Figure 1D). Despite this, alkaline pH_o_ still increased the rate of protein synthesis (Figure 1A).

### Increasing alkalinity activates insulin receptor family members in the heart

**Figure 2.**
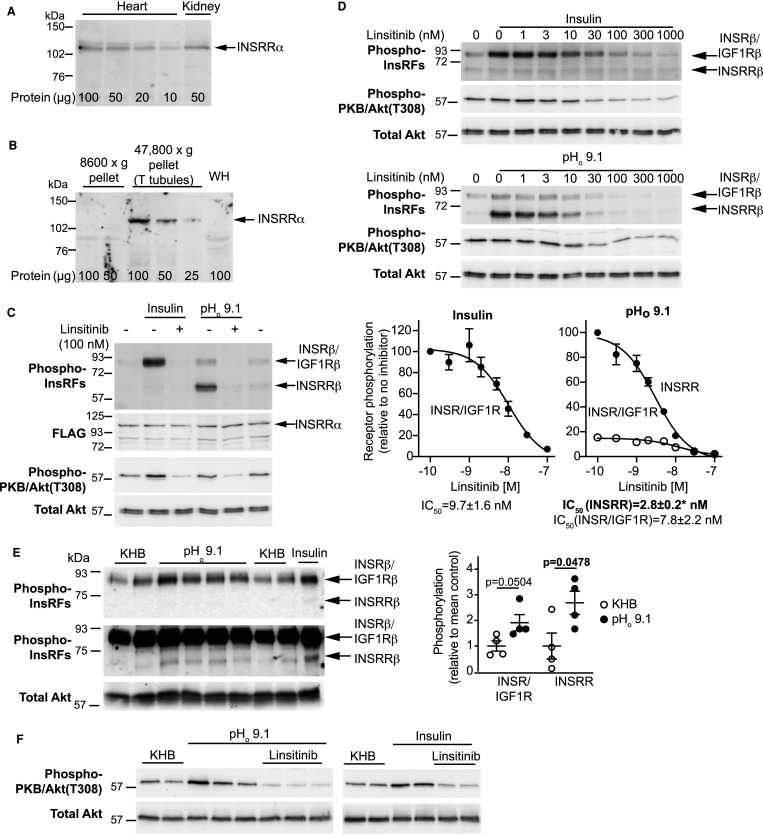
Alkaline pH_o_ activates insulin receptor family members (InsRFs) in the heart. (**A** and **B**) Rat heart or kidney whole extracts (**A**) or heart fractions from differential centrifugation (**B**) were immunoblotted for the INSRR α subunit. WH, whole heart. (**C** and **D**) HEK293 cells expressing human FLAG-INSRRs were treated (1 min) with insulin (50 mU/ml) or pH_o_ 9.1 buffer with/without the indicated concentrations of linsitinib. Samples were immunoblotted with antibodies to phosphorylated (Phospho-) InsRFs, FLAG or phospho- or total PKB/Akt. Blots are representative of three independent cell preparations. IC_50_ values for individual experiments were determined from densitometric data (**D**). Results are means ± SEM (*n* = 3). *P < 0.05 for IC_50_ for INSRR activated by alkaline pH_o_ relative to IC_50_ for the insulin receptor (INSR) activated by insulin (unpaired two-tailed *t*-test). (**E**) Male rat hearts were perfused (15 min) with Tris buffer at starting pH_o_ 9.1 or in Krebs Henseleit buffer (KHB) with/without 50 mU/ml insulin. Samples were immunoblotted with antibodies to phospho-InsRFs, or total PKB/Akt. Densitometric analysis is on the right as individual data points with means ± SEM (*n* = 4 per group). Statistical analysis used one-way ANOVA with Holm–Sidak post-test. Positions of relative molecular mass markers are on the left of the blots. (**F**) Activation of PKB/Akt by alkaline pH_o_ is mediated via InsRFs. Hearts were perfused as indicated with/without 1 µM linsitinib and samples were immunoblotted for phospho- or total PKB/Akt.

Insulin and IGF1 receptors (INSRs, IGF1Rs) are clearly important in the heart, but insulin receptor-related receptors (INSRRs) have not previously been studied. INSRRs are particularly highly expressed in the kidney where they are important in maintaining acid-base balance [16]. Immunoblotting with antibodies to the INSRR α subunit indicated that INSRRs are expressed in the heart at ∼50% the level in the kidney (Figure 2A), and are concentrated in membrane fractions enriched in T-tubules (Figure 2B). Previous studies indicated that INSRRs can form hybrid receptors with INSRs [25]. To confirm this, we overexpressed human INSRRs in HEK293 cells and assessed activation of INSRRs/INSRs/IGF1Rs by insulin and alkaline pH_o_ by immunoblotting for the highly conserved activating phosphorylations of the β subunit. The β subunit of INSRRs is smaller than that of INSRs and IGF1Rs [26] and is, therefore, distinguishable from the other receptors on immunoblots. As expected, insulin had a dominant effect on phosphorylation of insulin receptors, whilst pH_o_ 9.1 particularly increased phosphorylation of the overexpressed INSRRs (Figure 2C). However, pH_o_ 9.1 also increased phosphorylation of insulin/IGF1 receptors, suggesting that INSRRs can transactivate INSRs/IGF1Rs possibly in the context of hybrid receptors as in previous studies [25]. Linsitinib (OSI-906), a highly selective inhibitor of insulin receptor family kinases [18], inhibited phosphorylation of all insulin receptor family members and downstream activation of PKB/Akt by either insulin or pH_o_ 9.1 in HEK293 cells with overexpressed INSRRs (Figure 2C), being most potent at inhibiting INSRRs (Figure 2D). Endogenous INSRRs and INSRs/IGF1Rs were activated in perfused rat hearts by either insulin or alkaline pH_o_ (Figure 2E). The level of expression of endogenous INSRRs relative to INSRs/IGF1Rs in the heart is low (Figure 2E) in contrast to the relative levels of the receptors in HEK293 cells with overexpressed human INSRRs (Figure 2C). Nevertheless, the data suggest that hybrid receptors are present and functional in the heart. Activation of PKB/Akt in perfused hearts by insulin or alkaline pH_o_ was inhibited by linsitinib (Figure 2F). We conclude that alkaline pH_o_ activates insulin receptor family members in the heart, probably via INSRRs, and signals through PKB/Akt to increase protein synthesis.

### α_1_-adrenergic receptor agonists signal to PKB/Akt via insulin receptor family members to promote cardiomyocyte hypertrophy

**Figure 3.**
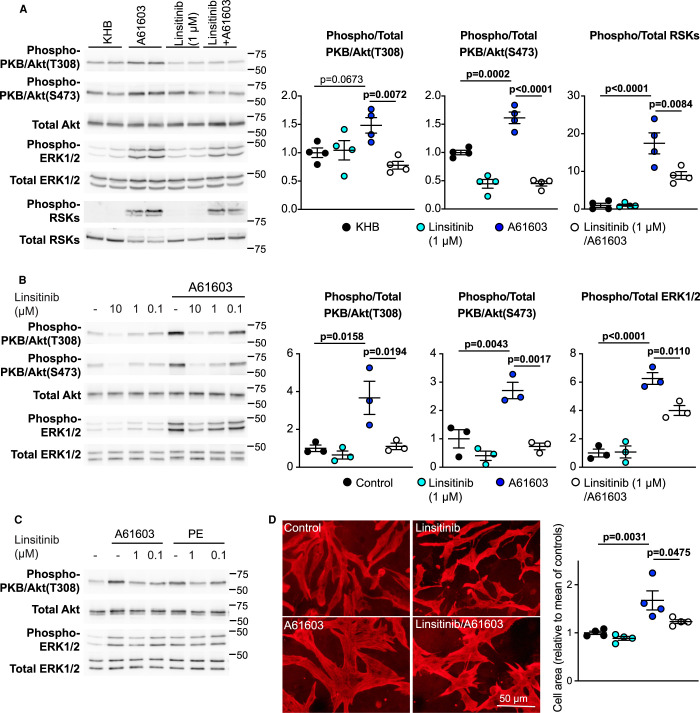
α_1_-ARs signal to PKB/Akt and ERK1/2 via insulin receptor family members. (**A**) Male rat hearts were perfused (30 min) with Krebs Henseleit Buffer (KHB) alone or with 50 nM A61603, with/without 1 µM linsitinib. Samples were immunoblotted for phosphorylated (Phospho-) or total kinases. Representative blots are on the left with densitometric analysis on the right. (**B** and **C**) Neonatal rat cardiomyocytes were untreated (control), or exposed to 50 nM A61603 or 100 µM phenylephrine (PE) for 5 min with/without the indicated concentrations of linsitinib. Samples were immunoblotted for phospho- or total kinases. In **B**, representative blots are on the left with densitometric analysis on the right. Individual data points are shown with means ± SEM. In **C**, a representative experiment is shown. (**D**) Neonatal rat cardiomyocytes were untreated (control), or exposed to 50 nM A61603 (24 h) with/without 1 µM linsitinib. Cells were immunostained with antibodies to troponin T. Images from a representative experiment are on the left with assessment of cell area on the right. Individual data points are shown with the means ± SEM (*n* = 3–4 per group). Statistical analysis used one-way ANOVA with Holm–Sidak post-test.

α_1_-AR agonists such as phenylephrine increase the rate of protein synthesis in perfused rat hearts and activate PKB/Akt in cardiomyocytes [9,27], but the mechanisms have not been established. We used linsitinib to determine if α_1_-ARs may signal via insulin receptor family members. The selective α_1A_-AR agonist A61603 activated PKB/Akt, ERK1/2 and p90RSKs in perfused hearts over 30 min (Figure 3A). Linsitinib prevented activation of PKB/Akt by A61603, indicating that PKB/Akt activation is mediated via insulin receptor family members. Activation of p90RSKs by A61603 was significantly reduced by linsitinib indicative of some contribution from the insulin receptor family to the ERK1/2 cascade in this context. Similarly, linsitinib suppressed activation of PKB/Akt and reduced activation of ERK1/2 by A61603 or phenylephrine in rat neonatal cardiomyocytes (Figure 3B,C). Moreover, it inhibited the increase in cardiomyocyte size induced by A61603, suggesting that insulin receptor family signalling is required for cardiomyocyte hypertrophy (Figure 3D). Thus, activation of PKB/Akt and, to some extent, ERK1/2 by α_1_-ARs requires insulin receptor family members, and this receptor crosstalk is necessary for cardiomyocyte hypertrophy.

### Linsitinib inhibits acute cardiac adaptation to phenylephrine *in vivo*, but subsequently exacerbates cardiac hypertrophy by increasing fibrosis

**Figure 4.**
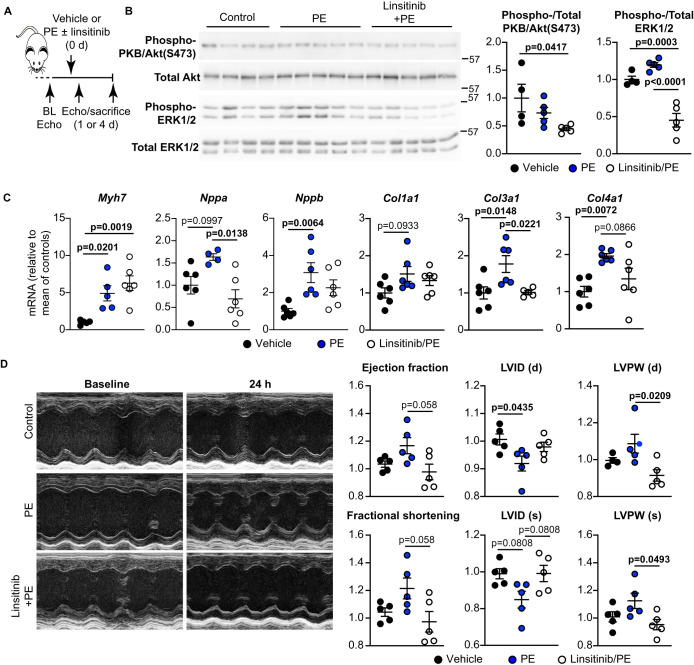
Linsitinib inhibits acute (24 h) activation of ERK1/2 and cardiac hypertrophy induced by PE *in vivo*. (**A**) Schematic of the study. C57Bl/6J male mice were subjected to baseline echocardiography (Echo) then implanted with minipumps to deliver vehicle only (Control), or phenylephrine (PE; 40 mg/kg/d) with/without linsitinib (2 mg/kg/d). Echocardiograms were taken at 1 or 4 days and mice were killed. (**B**) Heart samples (25 µg) were immunoblotted for phosphorylated (Phospho-) or total kinases. Representative blots are on the left with densitometric analysis on the right. (**C**) RNA was prepared from the mouse hearts and mRNA expression of hypertrophic (*Myh7, Nppa, Nppb*) and collagen (*Col1a1, Col3a1, Col4a1)* genes was assessed by qPCR. (**D**) Representative M-mode images (left) are shown at baseline and 24 h post minipump implantation, with quantitative analysis (right) of ejection fraction and fractional shortening, plus systolic (s) and diastolic (d) cardiac dimensions at 24 h relative to baseline measurements for individual mice. LVPW, left ventricular posterior wall; LVID, LV internal diameter. Individual data points are shown with means ± SEM (*n* = 5–6 per group). Statistical analysis used one-way ANOVA with Holm–Sidak's post-test.

α_1_-AR stimulation is generally associated with physiological adaptation of the heart and may prevent maladaptive remodelling [8]. We used linsitinib to determine if insulin receptor family members are potentially required for such adaptation *in vivo*. Studies in mice indicate 25–75 mg/kg/d linsitinib administered orally is effective against xenograft tumours [18]. The drug has 84–100% bioavailability, but a *t*_1/2_ of ∼2 h, so we selected a modest dose of 2 mg/kg/d (the equivalent expected after 10–12 h of oral dosing) for continuous delivery by osmotic minipumps. Adult male C57Bl/6J mice were treated with vehicle, linsitinib, phenylephrine (40 mg/kg/d) or linsitinib with phenylephrine, for 24 h to assess the effects on activation of PKB/Akt and ERK1/2, mRNA expression and the early adaptive response of the heart (assessed by echocardiography), and over 4 days to assess longer term consequences (Figure 4A). Mice receiving linsitinib alone, but not the other groups, had a significant increase in weight (26.33 ± 0.84 vs 25.86 ± 0.78 g, *P* = 0.049, repeated measures two-way ANOVA with Holm–Sidak's post-test; Supplementary Table S1). However, linsitinib alone did not significantly affect activation of PKB/Akt or ERK1/2, or modulate cardiac function or dimensions at 24 h or 4 days (Supplementary Figures S1 and S2, respectively).

Activation of PKB/Akt or ERK1/2 by phenylephrine in cardiomyocytes is transient and, although we detected increased ERK1/2 phosphorylation in the hearts from mice treated with phenylephrine for 24 h, we did not detect a significant change in PKB/Akt phosphorylation (Figure 4B). Nevertheless, linsitinib reduced the relative phosphorylation of both kinases in the presence of phenylephrine. At 24 h, phenylephrine increased mRNAs encoding the hypertrophic markers *Myh7*, *Nppa* and *Nppb*, in addition to the pro-fibrotic fibrillar collagens, *Col1a1 and Col3a1*, plus the basal lamina collagen, *Col4a1* (Figure 4C). Expression of all these genes apart from *Myh7* was reduced in the presence of linsitinib. Cardiac function/dimensions were assessed by echocardiography, comparing data obtained at 24 h with baseline data for individual animals. Echocardiography was performed on anaesthetised mice with anaesthesia maintained using 1.5% isoflurane. It should be noted that inhaled anaesthetics such as isoflurane transiently affect protein kinase signalling in the heart, including PI3K and PKB/Akt signalling [28], so we ensured that the same amount of anaesthetic was used for these studies. Echocardiography data are provided in Supplementary Table S4. Phenylephrine induced an early adaptive response with some increase in ejection fraction and fractional shortening, decreased left ventricular (LV) internal diameter and some increase in LV posterior wall thickness (Figure 4D). Treatment with linsitinib normalised all of these changes. These data suggest that insulin receptor family signalling is required for acute cardiac adaptation to α_1_-AR stimulation.

**Figure 5.**
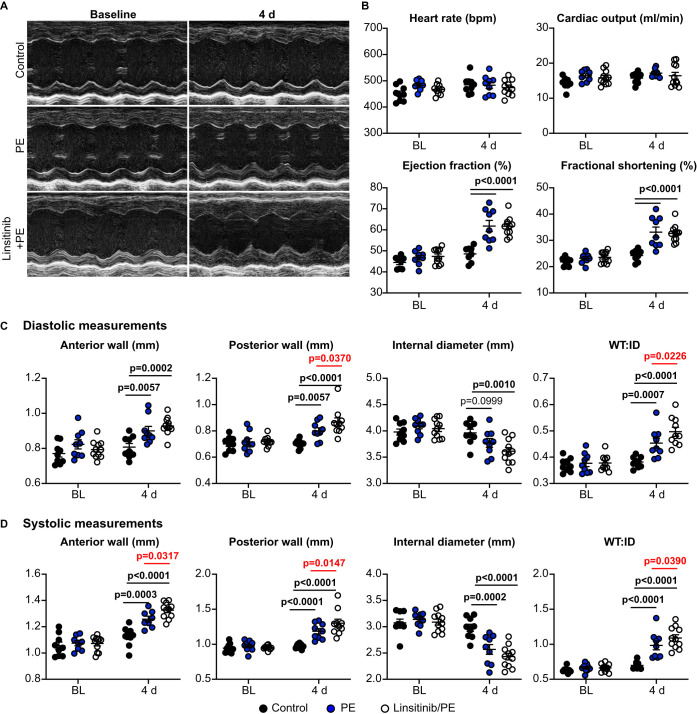
Linsitinib exacerbates cardiac hypertrophy induced by phenylephrine over 4 days. C57Bl/6J male mice were treated with vehicle alone (Control), or with phenylephrine (PE; 40 mg/kg/d) with/without linsitinib (2 mg/kg/d) using osmotic minipumps. Cardiac function/dimensions were assessed by echocardiography. (**A**) Representative M-mode images at baseline and 4 days post minipump implantation. Quantitative assessment of cardiac function (**B**) and left ventricular cardiac dimensions in diastole (**C**) and systole (**D**) at baseline (BL) and 4 days. WT:ID, ratio of wall thickness (WT = anterior wall + posterior wall) to internal diameter (ID). Individual data points are shown with means ± SEM (*n* = 7 per group). Statistical analysis used two-way ANOVA with Holm–Sidak's post-test.

**Figure 6.**
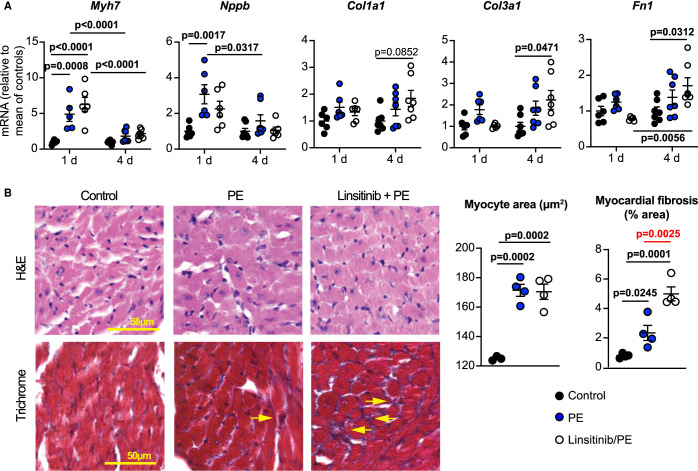
Linsitinib exacerbates cardiac fibrosis induced by phenylephrine *in vivo*. C57Bl/6J male mice were treated with vehicle alone (Control), or with phenylephrine (PE; 40 mg/kg/d) with/without linsitinib (2 mg/kg/d) for 4 days. (**A**) mRNA expression was measured by qPCR. Statistical analysis used two-way ANOVA with Holm–Sidak's post-test (1 day data are also shown in Figure 4 and are presented here for comparison). (**B**) Heart sections were stained for H&E (upper images) or Masson's Trichrome (lower images; yellow arrows indicate areas of fibrosis). Cardiomyocyte cross-sectional area and myocardial fibrosis were measured (right panels). Individual data points are shown with means ± SEM (*n* = 4–6 per group). Statistical analysis used one-way ANOVA with Holm–Sidak's post-test.

**Figure 7.**
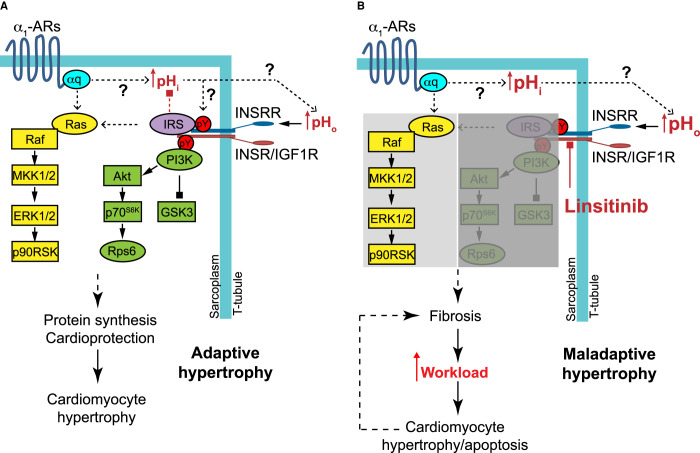
Schematic model of receptor cross-talk system from α_1_-adrenergic receptors (α_1_-ARs) via insulin receptor family members to cardiomyocyte hypertrophy. (**A**) In cardiomyocytes, α_1_-ARs signal through Gαq and increase intracellular pH_i_. Normalisation of pH_i_ increases extracellular pH_o_ which may trigger INSRRs in T tubules. This may serve to prevent excessive intracellular alkalinisation. Alternatively, INSRRs/INSRs/IGF1Rs may become phosphorylated via an intracellular mechanism. On activation, INSRRs/INSRs/IGF1Rs become Tyr phosphorylated and activated protein kinase B (PKB; also known as Akt), signalling via phosphoinositide 3′ kinase (PI3K; green). The extracellular signal-regulated kinase 1/2 (ERK1/2) cascade is also activated (yellow) partly via INSRRs/INSRs/IGF1Rs, but also through an alternative mechanism driven by Gαq. PKB/Akt and ERK1/2 signalling combine to promote protein synthesis, cardioprotection and adaptive hypertrophy. (**B**) Linsitinib inhibits INSRRs/INSRs/IGF1Rs, preventing PKB/Akt signalling and reducing ERK1/2 signalling, leading to increased fibrosis. This increases the workload of cardiomyocytes which hypertrophy, but the increased stress also causes cardiomyocyte apoptosis leading to further fibrosis and resulting in maladaptive hypertrophy. MKK, mitogen-activated protein kinase kinase; p90RSK, p90 ribosomal S6 kinase; IRS, insulin receptor substrate; GSK3, glycogen synthase kinase 3; p70S6K, p70 ribosomal S6 kinase.

We next assessed the consequences of linsitinib on cardiac adaptation to α_1_-AR stimulation over 4 days (Figure 5), when hypertrophic effects of phenylephrine are more clearly apparent whilst minimising longer term consequences of inhibiting insulin receptor signalling. At this time, phenylephrine significantly increased ejection fraction and fractional shortening and this was unaffected by linsitinib (Figure 5B). The degree of cardiac hypertrophy induced by phenylephrine increased between 24 h and 4 days, with increases in LV anterior and posterior wall thicknesses, decreased LV internal diameter and increased ratio of LV wall thickness to internal diameter (Figures 4D and 5C,D). Linsitinib significantly enhanced the degree of cardiac hypertrophy induced by phenylephrine at 4 days (Figure 5C,D). By 4 days, mRNA expression of pro-hypertrophic markers such as *Myh7* and *Nppb* that had been induced by phenylephrine at 24 h had essentially returned to baseline levels and this was unaffected by linsitinib (Figure 6A). In contrast, the small increases in expression of mRNAs for fibrillar collagens *Col1a1* and *Col3a1*, and other fibrotic genes such as *Fn1*, were enhanced by linsitinib. Linsitinib significantly increased the degree of interstitial fibrosis in hearts from mice treated with phenylephrine (Figure 6B), suggesting that insulin receptor family kinases are required for the development of compensated hypertrophy in the absence of fibrosis in the context of α_1_-AR stimulation. However, cardiomyocyte cross-sectional area was similar in mice receiving phenylephrine with or without linsitinib, so alternative hypertrophic mechanisms still operate. Potentially, cardiomyocyte hypertrophy in the presence of linsitinib is consequential to the increased workload resulting from increasing interstitial fibrosis. Nevertheless, the data indicate that insulin receptor family signalling is required for physiological adaptation of the heart to α_1_-AR stimulation because, in the absence of this, there is a shift to a more pathological response with enhanced fibrosis (Figure 7).

## Discussion

Heart failure is a major worldwide problem with a high and increasing cost to society. It is essential to find systems for protecting the heart following, for example, myocardial infarction, and so prevent progression from adaptive, compensated hypertrophy to decompensated hypertrophy and heart failure. One option is to harness existing, endogenous protective systems that operate in cardiomyocytes. This study identifies a novel signalling paradigm in cardiomyocytes with that potential (Figure 7). First, we established that relatively obscure INSRRs, related to other receptors well-known for their cardioprotective effects (INSRs/IGF1Rs), are not just expressed in the heart, but are functional and activate the same cardioprotective signalling pathways (Figure 2). Secondly, we demonstrated that a different receptor family, α_1_-ARs (also cardioprotective but which promote compensated hypertrophy [8,29]), signal via one or more of the insulin receptor family, and inhibiting the latter modulates cardiac adaptation to α_1_-AR agonists in cultured cells (Figure 3) and *in vivo* (Figures 4–6). Of ancillary importance is the observation that linsitinib, a drug developed to treat cancer, inhibited cardiac adaptation to α_1_-AR stimulation and increased interstitial fibrosis in even a very short period of time (Figures 4–6), although the drug alone did not appear to have any major effects (Supplemental Figures S1, S2). The data suggest that anti-cancer therapies that target insulin receptor family kinases may be cardiotoxic in some patients.

 Insulin and IGF1 receptors are known to play important roles in the heart. The third receptor of the family, the INSRR, is expressed in rat hearts (Figure 2A,B). Here, (as in kidney [16]) INSRRs are sensitive to extracellular alkaline conditions (Figure 2E,F) and, most likely, account for the observation that perfusion of rat hearts at alkaline pH_o_ promotes protein synthesis to the same degree as insulin [17]. INSRRs transactivated INSRs/IGF1Rs (Figure 2E), and alkaline pH_o_ increased the activating phosphorylations not just of INSRRs, but also INSRs/IGF1Rs. This receptor transactivation is potentially important since alkaline pH_o_ clearly activates PKB/Akt (Figures 1B and 2F), yet INSRR β subunits are truncated at the C-terminus, lacking the recognised docking site for PI3K [30] required for PKB/Akt activation. Potentially, the signal is initiated via INSRs/IGF1Rs following receptor transactivation. Our results also indicate that α_1_-ARs transactivate insulin receptor family members to promote cardiomyocyte hypertrophy (Figure 7). Cross-talk from GqPCRs to receptor protein tyrosine kinases is not a new concept. For example, angiotensin II transactivates epidermal growth factor (EGF) receptors, a mechanism involving activation of a matrix metalloproteinase that releases Hb-EGF extracellularly that then binds to and activates EGF receptors [31]. Our data for α_1_-AR signalling provides an important link from a receptor known to promote compensated hypertrophy to the increase in protein synthesis required for that hypertrophy to occur, operating through an established receptor system renowned for its potent effects on protein synthesis.

INSRRs require pH_o_ > 8.0 for activation [16], and the kidney (the only tissue where INSRRs have so far been studied in detail) is clearly involved with maintenance of acid-base balance with the potential for the receptors to become activated by such pH levels. Since blood pH is maintained at 7.35–7.45 and an increase to just pH 7.6 is associated with high mortality [32], the relevance of an alkali-sensor in the heart is not immediately obvious. INSRRs (or any of the insulin receptor family) could be activated intracellularly by tyrosine phosphorylation of the intracellular kinase domain by non-receptor tyrosine kinases (e.g. Src family kinases [33]) or by low level activity of the receptors themselves coupled with inhibition of a phosphatase. Indeed, INSRs, IGF1Rs and INSRRs are all activated by the insulinomimetic combination of oxidative stress and vanadate which inhibit tyrosine phosphatases facilitating Tyr phosphorylation [26]. Nevertheless, INSRRs in the heart may serve to boost the cardioprotective response, perhaps supplementing insulin/IGF1 signalling, and cardiac expression of INSRRs may have no bearing on external alkalinity. However, INSRRs appear to be concentrated in a membrane fraction enriched in the transverse T tubules (Figure 2B). These small invaginations are designed to facilitate rapid contraction/relaxation, being enriched in ion channels. They are increasingly recognised to form signalling ‘hubs', and the small volumes restrict access to the extracellular fluids resulting in microdomains of ions [34,35]. In this environment, localised changes in ion balance may generate a sufficiently high pH_o_ to activate INSRRs.

Cardiac contractility is heavily influenced by intracellular pH_i_ and, for example, increased pH_i_ enhances calcium sensitivity of the myofilaments, increasing force of contraction and rate of relaxation [36,37]. Maintaining intracellular pH_i_ in cardiomyocytes within a normal range is therefore of vital importance. α_1_-AR agonists such as phenylephrine increase intracellular pH_i_ in rat and cat cardiomyocytes by 0.1–0.2 pH unit and this contributes to its positive inotropic effect [38,39]. Similarly, angiotensin II increases intracellular pH_i_ in rabbit cardiomyocytes [40]. One of the main systems for balancing intracellular alkalinisation of cardiomyocytes is via anion exchanger 3 (AE3, a Cl^−^/HCO_3_^−^ exchanger that extrudes HCO_3_^−^ from the cell), coupled to an intracellular carbonic anhydrase (e.g. CAII) that generates the HCO_3_^−^[41,42]. The rate of recovery of pH_i_ from intracellular alkalosis is much reduced in AE3^−/−^ cardiomyocytes, confirming that AE3 plays an important role in balancing pH_i_, and α_1_-AR-induced cardiomyocyte hypertrophy is abolished in AE3^−/−^ cardiomyocytes [43]. Carbonic anhydrase inhibitors and knockout of CAII also prevent α_1_-AR induced cardiomyocyte hypertrophy [44,45]. Thus, the CAII/AE3 system is necessary to extrude HCO_3_^-^ from cardiomyocytes following stimulation by α_1_-AR agonists for hypertrophy to occur. The presence of AE3 in the confines of T tubules [46] has potential to increase pH_o_ in close proximity to INSRRs that simultaneously confer a high degree of cardioprotection. Clearly, further work is required to determine whether α_1_-ARs activate insulin receptor family kinases via an intracellular or extracellular system. Further work is also required to establish whether INSRRs operate to modulate the increase in intracellular pH_i_ that develops in cardiomyocytes following exposure to α_1_-AR agonists [38,39].

Irrespective of the actual process of the crosstalk, our data (establishing that α_1_-ARs signal via INSRs/IGF1Rs/INSRRs to elicit cardioprotective signalling via PKB/Akt) potentially increases the therapeutic repertoire for exploitation. It is already known that activation of α_1_-ARs *per se* is cardioprotective [8,29], and that insulin or IGF1 activation of their receptors is also cardioprotective [47–49]. However, the effects of α_1_-ARs to increase intracellular pH_i_ [38,39] may not always be desirable and, because of the pleiotropic effects of insulin and IGF1 throughout the body, activating INSRs/IGF1s to protect the heart is also potentially undesirable unless in the very short term. In contrast, INSRR expression is more restricted, with receptors confined to specific subdomains within certain cells and selected tissues, so activation of these receptors for cardioprotection may be a more viable way to harness the pathway for therapeutic purposes. Developing synthetic agonists to target insulin receptor family members is not an easy task and even a substitute for insulin has proved difficult to produce. However, as in cancer, one therapeutic option might be an activating monoclonal antibody to target INSRRs, at least one of which has previously been produced [50]. Peptide mimetics may prove an alternative viable option.

This study developed from observations reported in 1989 on the effects of perfusion of rat hearts with alkaline pH_o_ buffers [17], but other questions remain from that era and before. For example, the heart utilises long chain fatty acids as a fuel source and switches between glucose and fatty acids to maintain energy levels for contraction [51]. Non-carbohydrate fuels (long chain fatty acids, ketone bodies, lactate, pyruvate, acetate) also stimulate protein synthesis in the glucose-perfused heart to values comparable to those observed with insulin [52–54]. Although the effects of amino acids on mTOR signalling and protein synthesis have been explored [55], as far as we are aware, there is still little understanding of how non-carbohydrate fuels regulate cellular functions such as protein synthesis in the heart. Long-chain fatty acids influence insulin signalling (e.g. palmitate counteracts the inhibition of AMPK by insulin and the recovery of contractile power following ischaemia–reperfusion [56]), and further studies are necessary to understand the relationships between fuel sources, signalling pathways and cellular processes in the heart.

There are other situations in which INSRRs may play a significant role. For example, pressure-overload on the heart induced by thoracic aortic banding (TAC) is associated with enhanced insulin receptor signalling with increased phosphorylation of INSR/IGF1R and PKB/Akt [57]. INSRRs could be involved in this response, potentially being activated as a consequence of the developing disease and possibly being up-regulated. Interestingly, this particular study presented evidence to suggest that insulin receptor signalling may contribute to maladaptive hypertrophy. Another situation in which PKB/Akt is activated in the heart and for which the mechanism remains relatively unresolved is in the context of ischaemia/reperfusion (as occurs in myocardial infarction) [58]. It is well-established that ischaemia causes intracellular acidosis. Reperfusion (required to restore cardiac function even though it causes reperfusion injury) is associated with rapid normalisation of intracellular pH_i_ associated with various changes in ion fluxes [59], which could impact on INSRR signalling. Reperfusion also results in a high level of oxidative stress, known to activate PKB/Akt in cardiomyocytes [60]. This may involve inhibition of tyrosine phosphatases, but the mechanisms are not fully resolved. Clearly, further studies would be useful to establish if INSRRs are involved in such events and to address the question of when/if insulin receptor signalling in cardiomyocytes can be maladaptive.

The main focus of our study was the PKB/Akt signalling pathway, given its involvement in cytoprotection and increasing protein synthesis [12]. ERK1/2 signalling is also protective and promotes protein synthesis, and was activated in rat hearts perfused at alkaline pH_o_ (Figure 1D), potentially contributing to the increase in protein synthesis induced by these conditions. It seems unlikely that ERK1/2 activation was mediated via INSRs/IGF1Rs, since insulin did not activate ERK1/2 to any significant degree in perfused hearts (Figure 1D), and neither insulin nor IGF1 activate ERK1/2 in cardiomyocytes [24]. Insulin activates ERK1/2 in other systems, most likely via IRS proteins [61], and it is possible that the compartmentalisation associated with cardiomyocytes (most clearly illustrated in relation to β-adrenergic receptors and cAMP signalling [62]) precludes ERK1/2 activation by insulin in these cells. How, then, are ERK1/2 activated in the heart by alkaline pH_o_? JNKs and p38-MAPKs were activated simultaneously with ERK1/2 (Figure 1D), and activation of all three may be a consequence of the stress on the heart resulting from increased extracellular pH_o_ that will impact on intracellular pH_i_. Upstream signalling events leading to MAPK activation in response to stresses remain relatively under-investigated in cardiomyocytes, but could involve, for example, Rho family small G proteins and or any of a number of MKK kinases [3]. Alternatively, inhibition of phosphatases required to keep the pathways under control could also result in pathway activation. Clearly, any combination of these various factors may be involved, and further studies are necessary to determine the precise mechanisms.

PKB/Akt and ERK1/2 are both implicated in cardiac hypertrophy with many studies simply assessing their activation under various conditions in different systems. This is justifiable because they each form a nexus where signals become integrated from different stimuli, whilst divergent signalling downstream elicits different responses. Various substrates of PKB/Akt and ERK1/2 have been identified, some of which are clearly implicated in cardiac pathophysiology. For example, phosphorylation and inhibition of GSK3α/β by PKB/Akt is a key element in the development of cardiac hypertrophy [63]. For ERK1/2, key substrates include p90RSKs (which regulate the Na^+^/H^+^ exchanger [64] and modulate gene expression [7]), in addition to transcription factors [3]. It should perhaps be considered that the overall response of the heart, whether adaptive or maladaptive hypertrophy, is a reflection of how these downstream divergent signals become integrated both at the level of individual cells (cardiomyocytes, cardiac fibroblasts, endothelial cells) that may hypertrophy or proliferate, and the heart as a whole. Developing this understanding represents a major challenge that will most likely require application of mathematical modelling to the various intra- and intercellular signalling components.

There are, of course, limitations to our study. Probably the most important relates to our proposal that INSRRs are the key mediators of the cardiac response to alkaline pH_o_ and these form a potential therapeutic target for cardioprotection (Figure 7). This is based largely on the use of a single inhibitor, linsitinib, albeit one which is highly selective for the insulin receptor family kinases. Further exploration and validation will be necessary to dissect the role of INSRRs in the heart. Initial studies may need to rely on the use of genetically modified mice such as the INSRR knockout mouse (as in [16]) or, preferably, a system designed for conditional gene deletion of INSRRs in cardiomyocytes. Further studies may benefit from development of specific receptor antagonists or agonists as discussed above. A second consideration is that we only assessed the acute effects of linsitinib on phenylephrine-induced hypertrophy, taking the study to just 4 d. From the perspective of effects of anti-cancer agents and potential cardiotoxicity, further studies are necessary over a longer time-frame to establish whether the pathology stabilises or if there is decompensation and development of heart failure. The echocardiograms provided data on the changes in cardiac dimensions with information on systolic cardiac function (Figures 4 and 5). However, we did not assess diastolic function and, given the effects of linsitinib on cardiac fibrosis, this is an important consideration for longer-term studies that could lend insight into development of heart failure with preserved ejection fraction (HFpEF) [65].

In summary, our data identify a novel receptor (the INSRR) and system of receptor cross-talk (from α_1_-ARs to the insulin receptor family) in cardiomyocytes (Figure 7). In our working model, α_1_-ARs signal through Gαq and increase intracellular pH_i_, normalisation of which increases extracellular pH_o_ which may trigger INSRRs in T tubules. This may serve to prevent excessive intracellular alkalinisation. Alternatively, INSRRs/INSRs/IGF1Rs may become phosphorylated via an intracellular mechanism. On activation, INSRRs/INSRs/IGF1Rs become Tyr phosphorylated and activate PKB/Akt signalling via PI3K. The ERK1/2 cascade is activated partly via INSRRs/INSRs/IGF1Rs, but also through an alternative mechanism driven by Gαq. PKB/Akt and ERK1/2 signalling combine to promote protein synthesis, cardioprotection and adaptive hypertrophy. Anti-cancer drugs that target insulin receptor family kinases (such as linsitinib) inhibit INSRRs/INSRs/IGF1Rs, preventing PKB/Akt signalling and reducing ERK1/2 signalling. This leads to increased fibrosis and, potentially, maladaptive hypertrophy. We suggest that INSRRs constitute a novel therapeutic modality for cardioprotection and understanding more of the crosstalk mechanism may facilitate the identification of further therapeutic options.

## Data Availability

All primary data are available from the corresponding author upon reasonable request. Additional data sharing information is not applicable to this study.

## Open Access

Open access for this article was enabled by the participation of University of Reading in an all-inclusive *Read & Publish* pilot with Portland Press and the Biochemical Society under a transformative agreement with JISC.

## Abbreviations

AR: adrenergic receptor
BSA: bovine serum albumin
DMEM: Dulbecco's modified Eagle's medium
DMSO: dimethyl sulphoxide
EDTA: ethylene diamine tetraacetic acid
EGF: epidermal growth factor
ERK: extracellular signal-regulated kinase
GqPCR: Gq protein-coupled receptor
GSK: glycogen synthase kinase
IGF1: insulin-like growth factor 1
IGF1R: IGF1 receptor
INSR: insulin receptor
INSRR: insulin receptor-related receptor
InsRF: insulin receptor family
JNK: c-Jun N-terminal kinase
KHBBS: Krebs-Henseleit bicarbonate-buffered saline
LV: left ventricular
MAPK: mitogen-activated protein kinase
MKK: MAPK kinase
mTOR: mammalian target of rapamycin
p70S6K: p70 S6 kinase
p90RSK: p90 ribosomal S6 kinase
PE: phenylephrine
PI3K: phosphoinositide 3′ kinase
PKB: protein kinase B

## References

[1] Dorn, G.W., Robbins, J. and Sugden, P.H. (2003) Phenotyping hypertrophy: eschew obfuscation. Circ. Res. 92, 1171–1175 10.1161/01.RES.0000077012.11088.BC 12805233

[2] Nakamura, M. and Sadoshima, J. (2018) Mechanisms of physiological and pathological cardiac hypertrophy. Nat. Rev. Cardiol. 15, 387–407 10.1038/s41569-018-0007-y 29674714

[3] Clerk, A., Cullingford, T.E., Fuller, S.J., Giraldo, A., Markou, T., Pikkarainen, S. et al. (2007) Signaling pathways mediating cardiac myocyte gene expression in physiological and stress responses. J. Cell Physiol. 212, 311–322 10.1002/jcp.21094 17450511

[4] Matsui, T. and Rosenzweig, A. (2005) Convergent signal transduction pathways controlling cardiomyocyte survival and function: the role of PI 3-kinase and Akt. J. Mol. Cell Cardiol. 38, 63–71 10.1016/j.yjmcc.2004.11.005 15623422

[5] Roux, P.P. and Topisirovic, I. (2018) Signaling pathways involved in the regulation of mRNA translation. Mol. Cell. Biol. 38, e00070-18 10.1128/MCB.00070-18 29610153 PMC5974435

[6] Markou, T., Marshall, A.K., Cullingford, T.E., Tham, E.L., Sugden, P.H. and Clerk, A. (2010) Regulation of the cardiomyocyte transcriptome vs translatome by endothelin-1 and insulin: translational regulation of 5′ terminal oligopyrimidine tract (TOP) mRNAs by insulin. BMC Genomics 11, 343 10.1186/1471-2164-11-343 20509958 PMC2900265

[7] Amirak, E., Fuller, S.J., Sugden, P.H. and Clerk, A. (2013) p90 ribosomal S6 kinases play a significant role in early gene regulation in the cardiomyocyte response to Gq protein-coupled receptor stimuli, endothelin-1 and α_1_-adrenergic receptor agonists. Biochem. J. 450, 351–363 10.1042/BJ20121371 23215897 PMC3573779

[8] O'Connell, T.D., Jensen, B.C., Baker, A.J. and Simpson, P.C. (2014) Cardiac α_1_-adrenergic receptors: novel aspects of expression, signaling mechanisms, physiologic function, and clinical importance. Pharmacol. Rev. 66, 308–333 10.1124/pr.112.007203 24368739 PMC3880467

[9] Clerk, A. and Sugden, P.H. (1999) Activation of protein kinase cascades in the heart by hypertrophic G protein-coupled receptor agonists. Am. J. Cardiol. 83, 64H–69H 10.1016/S0002-9149(99)00261-1 10750590

[10] Cullingford, T.E., Markou, T., Fuller, S.J., Giraldo, A., Pikkarainen, S., Zoumpoulidou, G. et al. (2008) Temporal regulation of expression of immediate early and second phase transcripts by endothelin-1 in cardiomyocytes. Genome Biol. 9, R32 10.1186/gb-2008-9-2-r32 18275597 PMC2374717

[11] Rose, B.A., Force, T. and Wang, Y. (2010) Mitogen-activated protein kinase signaling in the heart: angels versus demons in a heart-breaking tale. Physiol. Rev. 90, 1507–1546 10.1152/physrev.00054.2009 20959622 PMC3808831

[12] Lawlor, M.A. and Alessi, D.R. (2001) PKB/akt: a key mediator of cell proliferation, survival and insulin response? J. Cell Sci. 114, 2903–2910 10.1242/jcs.114.16.2903 11686294

[13] Hubbard, S.R. (2013) The insulin receptor: both a prototypical and atypical receptor tyrosine kinase. Cold Spring Harb. Perspect. Biol. 5, a008946 10.1101/cshperspect.a008946 23457259 PMC3578362

[14] Catalucci, D., Latronico, M.V.G., Ellsingen, O. and Condorelli, G. (2008) Physiological hypertrophy: how and why? Front. Biosci. 13, 312–324 10.2741/2681 17981549

[15] Shier, P. and Watt, V.M. (1989) Primary structure of a putative receptor for a ligand of the insulin family. J. Biol. Chem. 264, 14605–14608 10.1016/S0021-9258(18)63737-8 2768234

[16] Deyev, I.E. Sohet, F., Vassilenko, K.P., Serova, O.V., Popova, N.V., Zozulya, S.A. et al. (2011) Insulin receptor-related receptor as an extracellular alkali sensor. Cell Metab. 13, 679–689 10.1016/j.cmet.2011.03.022 21641549 PMC3119365

[17] Fuller, S.J., Gaitanaki, C.J. and Sugden, P.H. (1989) Effects of increasing extracellular pH on protein synthesis and protein degradation in the perfused working rat heart. Biochem. J. 259, 173–179 10.1042/bj2590173 2719641 PMC1138488

[18] Mulvihill, M.J., Cooke, A., Rosenfeld-Franklin, M., Buck, E., Foreman, K., Landfair, D. et al. (2009) Discovery of OSI-906: a selective and orally efficacious dual inhibitor of the IGF-1 receptor and insulin receptor. Future Med. Chem. 1, 1153–1171 10.4155/fmc.09.89 21425998

[19] Sugden, P.H. and Fuller, S.J. (1991) Correlations between cardiac protein synthesis rates, intracellular pH and the concentrations of creatine metabolites. Biochem. J. 273, 339–346 10.1042/bj2730339 1991035 PMC1149851

[20] Bogoyevitch, M.A., Gillespie-Brown, J., Ketterman, A.J., Fuller, S.J., Ben-Levy, R., Ashworth, A. et al. (1996) Stimulation of the stress-activated mitogen-activated protein kinases subfamilies in perfused heart. p38/RK mitogen-activated kinases and c-Jun N-terminal kinases are activated by ischemia-reperfusion. Circ. Res. 79, 162–173 10.1161/01.RES.79.2.162 8755992

[21] Tishkoff, D.X., Nibbelink, K.A., Holmberg, K.H., Dandu, L. and Simpson, R.U. (2008) Functional vitamin D receptor (VDR) in the t-tubules of cardiac myocytes: VDR knockout cardiomyocyte contractility. Endocrinology 149, 558–564 10.1210/en.2007-0805 17974622 PMC2219302

[22] Marshall, A.K., Barrett, O.P., Cullingford, T.E., Shanmugasundram, A., Sugden, P.H. and Clerk, A. (2010) ERK1/2 signaling dominates over RhoA signaling in regulating early changes in RNA expression induced by endothelin-1 in neonatal rat cardiomyocytes. PLoS ONE 5, e10027 10.1371/journal.pone.0010027 20368814 PMC2848868

[23] Proud, C.G. (2004) Ras, PI3-kinase and mTOR signaling in cardiac hypertrophy. Cardiovasc. Res. 63, 403–413 10.1016/j.cardiores.2004.02.003 15276465

[24] Clerk, A., Aggeli, I.K.S., Stathopoulou, K. and Sugden, P.H. (2006) Peptide growth factors signal differentially through protein kinase C to extracellular signal-regulated kinases in neonatal cardiomyocytes. Cell Signal. 18, 225–235 10.1016/j.cellsig.2005.04.005 15936927

[25] Jui, H.Y., Accili, D. and Taylor, S.I. (1996) Characterization of a hybrid receptor formed by dimerization of the insulin receptor-related receptor (IRR) with the insulin receptor (IR): coexpression of cDNAs encoding human IRR and human IR in NIH-3T3 cells. Biochemistry 35, 14326–14330 10.1021/bi9613032 8916919

[26] Jui, H.Y., Suzuki, Y., Accili, D. and Taylor, S.I. (1994) Expression of a cDNA encoding the human insulin receptor-related receptor. J. Biol. Chem. 269, 22446–22452 10.1016/S0021-9258(17)31810-0 8071374

[27] Fuller, S.J., Gaitanaki, C.J. and Sugden, P.H. (1990) Effects of catecholamines on protein synthesis in cardiac myocytes and perfused hearts isolated from adult rats. stimulation of translation is mediated through the α1-adrenoceptor. Biochem. J. 266, 727–736 10.1042/bj2660727 1970237 PMC1131200

[28] Raphael, J., Rivo, J. and Gozal, Y. (2005) Isoflurane-induced myocardial preconditioning is dependent on phosphatidylinositol-3-kinase/Akt signalling. Br. J. Anaesth. 95, 756–763 10.1093/bja/aei264 16286350

[29] O'Connell, T.D., Swigart, P.M., Rodrigo, M.C., Ishizaka, S., Joho, S., Turnbull, L. et al. (2006) α_1_-Adrenergic receptors prevent a maladaptive response to pressure overload. J. Clin. Invest. 116, 1005–1015 10.1172/JCI22811 16585965 PMC1421341

[30] Hanke, S. and Mann, M. (2009) The phosphotyrosine interactome of the insulin receptor family and its substrates IRS-1 and IRS-2. Mol. Cell Proteomics 8, 519–534 10.1074/mcp.M800407-MCP200 19001411 PMC2649814

[31] Shah, B.H. and Catt, K.J. (2003) A central role of EGF receptor transactivation in angiotensin II-induced cardiac hypertrophy. Trends Pharmacol. Sci. 24, 239–244 10.1016/S0165-6147(03)00079-8 12767723

[32] Anderson, L.E. and Henrich, W.L. (1987) Alkalemia-associated morbidity and mortality in medical and surgical patients. South Med. J. 80, 729–733 10.1097/00007611-198706000-00016 3589765

[33] Amatya, N., Lin, D.Y. and Andreotti, A.H. (2019) Dynamic regulatory features of the protein tyrosine kinases. Biochem. Soc. Trans. 47, 1101–1116 10.1042/BST20180590 31395755 PMC7285797

[34] Hong, T. and Shaw, R.M. (2017) Cardiac T-tubule microanatomy and function. Physiol. Rev. 97, 227–252 10.1152/physrev.00037.2015 27881552 PMC6151489

[35] Ibrahim, M., Gorelik, J., Yacoub, M.H. and Terracciano, C.M. (2011) The structure and function of cardiac t-tubules in health and disease. Proc. Biol. Sci. 278, 2714–2723 10.1098/rspb.2011.0624 21697171 PMC3145195

[36] Kim, D. and Smith, T.W. (1987) Altered Ca fluxes and contractile state during pH changes in cultured heart cells. Am. J. Physiol. 253, C137–C146 10.1152/ajpcell.1987.253.1.C137 3605326

[37] Spitzer, K.W. and Bridge, J.H. (1992) Relationship between intracellular pH and tension development in resting ventricular muscle and myocytes. Am. J. Physiol. 262, C316–C327 10.1152/ajpcell.1992.262.2.C316 1539624

[38] Terzic, A., Pucéat, M., Clément, O., Scamps, F. and Vassort, G. (1992) α_1_-Adrenergic effects on intracellular pH and calcium and on myofilaments in single rat cardiac cells. J. Physiol. 447, 275–292 10.1113/jphysiol.1992.sp019002 1317431 PMC1176036

[39] Vila-Petroff, M., Pérez, G.N., Alvarez, B., Cingolani, H.E. and Mattiazzi, A. (1996) Mechanism of negative lusitropic effect of α_1_-adrenoceptor stimulation in cat papillary muscles. Am. J. Physiol. 270, H701–H709 10.1152/ajpheart.1996.270.2.H701 8779848

[40] Ikenouchi, H., Barry, W.H., Bridge, J.H.B., Weinberg, E.O., Apstein, C.S. and Lorell, B.H. (1994) Effects of angiotensin II on intracellular Ca^2+^ and pH in isolated beating rabbit hearts and in myocytes loaded with the indicator indo-1. J. Physiol. 480, 203–215 10.1113/jphysiol.1994.sp020353 7869240 PMC1155839

[41] Casey, J.R., Grinstein, S. and Orlowski, J. (2010) Sensors and regulators of intracellular pH. Nat. Rev. Mol. Cell Biol. 11, 50–61 10.1038/nrm2820 19997129

[42] Wang, H.S., Chen, Y., Vairamani, K. and Shull, G.E. (2014) Critical role of bicarbonate and bicarbonate transporters in cardiac function. World J. Biol. Chem. 5, 334–345 10.4331/wjbc.v5.i3.334 25225601 PMC4160527

[43] Sowah, D., Brown, B.F., Quon, A., Alvarez, B.V. and Casey, J.R. (2014) Resistance to cardiomyocyte hypertrophy in ae3-/- mice, deficient in the AE3 Cl-/HCO3- exchanger. BMC Cardiovasc. Disord. 14, 89 10.1186/1471-2261-14-89 25047106 PMC4120010

[44] Alvarez, B.V., Johnson, D.E., Sowah, D., Soliman, D., Light, P.E., Xia, Y. et al. (2007) Carbonic anhydrase inhibition prevents and reverts cardiomyocyte hypertrophy. J. Physiol. 579, 127–145 10.1113/jphysiol.2006.123638 17124262 PMC2075384

[45] Brown, B.F., Quon, A., Dyck, J.R. and Casey, J.R. (2012) Carbonic anhydrase II promotes cardiomyocyte hypertrophy. Can. J. Physiol. Pharmacol. 90, 1599–1610 10.1139/y2012-142 23210439

[46] Pucéat, M., Korichneva, I., Cassoly, R. and Vassort, G. (1995) Identification of band 3-like proteins and Cl-/HCO3- exchange in isolated cardiomyocytes. J. Biol. Chem. 270, 1315–1322 10.1074/jbc.270.3.1315 7836397

[47] Baines, C.P., Wang, L., Cohen, M.V. and Downey, J.M. (1999) Myocardial protection by insulin is dependent on phospatidylinositol 3-kinase but not protein kinase C or K_ATP_ channels in the isolated rabbit heart. Basic Res. Cardiol. 94, 188–198 10.1007/s003950050142 10424237

[48] Jonassen, A.K., Sack, M.N., Mjos, O.D. and Yellon, D.M. (2001) Myocardial protection by insulin at reperfusion requires early administration and is mediated by Akt and p70s6 kinase cell-survival signaling. Circ. Res. 89, 1191–1198 10.1161/hh2401.101385 11739285

[49] Duerr, R.L., McKirnan, M.D., Gim, R.D., Clark, R.G., Chien, K.R. and Ross, Jr, J. (1996) Cardiovascular effects of insulin-like growth factor-1 and growth hormone in chronic left ventricular failure in the rat. Circulation 93, 2188–2196 10.1161/01.CIR.93.12.2188 8925588

[50] Kovacina, K.S. and Roth, R.A. (1995) Characterization of the endogenous insulin receptor-related receptor in neuroblastomas. J. Biol. Chem. 270, 1881–1887 10.1074/jbc.270.4.1881 7829525

[51] Glatz, J.F.C., Nabben, M., Young, M.E., Schulze, P.C., Taegtmeyer, H. and Luiken, J.J.F.P. (2020) Re-balancing cellular energy substrate metabolism to mend the failing heart. Biochim. Biophys. Acta Mol. Basis Dis. 1866, 165579 10.1016/j.bbadis.2019.165579 31678200 PMC7586321

[52] Rannels, D.E., Hjalmarson, A.C. and Morgan, H.E. (1974) Effects of noncarbohydrate fuels on protein synthesis in muscle. Am. J. Physiol. 226, 528–539 10.1152/ajplegacy.1974.226.3.528 4817404

[53] Kochel, P.J., Kira, Y., Gordon, E.E. and Morgan, H.E. (1984) Effects of noncarbohydrate substrates on protein synthesis in hearts from fed and fasted rats. J. Mol. Cell Cardiol. 16, 371–383 10.1016/S0022-2828(84)80608-2 6374161

[54] Smith, D.M., Fuller, S.J. and Sugden, P.H. (1986) The effects of lactate, acetate, glucose,insulin,starvation and alloxan-diabetes on protein synthesis in perfused rat hearts. Biochem. J. 236, 543–547 10.1042/bj2360543 3530250 PMC1146874

[55] Condon, K.J. and Sabatini, D.M. (2019) Nutrient regulation of mTORC1 at a glance. J. Cell Sci. 132, jcs222570 10.1242/jcs.222570 31722960 PMC6857595

[56] Folmes, C.D., Clanachan, A.S. and Lopaschuk, G.D. (2006) Fatty acids attenuate insulin regulation of 5'-AMP-activated protein kinase and insulin cardioprotection after ischemia. Circ. Res. 99, 61–68 10.1161/01.RES.0000229656.05244.11 16741157

[57] Shimizu, I., Minamino, T., Toko, H., Okada, S., Ikeda, H., Yasuda, N. et al. (2010) Excessive cardiac insulin signaling exacerbates systolic dysfunction induced by pressure overload in rodents. J. Clin. Invest. 120, 1506–1514 10.1172/JCI40096 20407209 PMC2860916

[58] Hausenloy, D.J. and Yellon, D.M. (2004) New directions for protecting the heart against ischaemia- reperfusion injury: targeting the reperfusion injury salvage kinase (RISK)-pathway. Cardiovasc. Res. 61, 448–460 10.1016/j.cardiores.2003.09.024 14962476

[59] Turer, A.T. and Hill, J.A. (2010) Pathogenesis of myocardial ischemia-reperfusion injury and rationale for therapy. Am. J. Cardiol. 106, 360–368 10.1016/j.amjcard.2010.03.032 20643246 PMC2957093

[60] Pham, F.H., Sugden, P.H. and Clerk, A. (2000) Regulation of protein kinase B and 4E-BP1 by oxidative stress in cardiac myocytes. Circ. Res. 86, 1252–1258 10.1161/01.RES.86.12.1252 10864916

[61] Withers, D.J. and White, M. (2000) The insulin signaling system - a common link in the pathogenesis of type 2 diabetes. Endocrinology 141, 1917–1921 10.1210/endo.141.6.7584 10830270

[62] Gorelik, J., Wright, P.T., Lyon, A.R. and Harding, S.E. (2013) Spatial control of the βAR system in heart failure: the transverse tubule and beyond. Cardiovasc. Res. 98, 216–224 10.1093/cvr/cvt005 23345264 PMC3633155

[63] Lal, H., Ahmad, F., Woodgett, J. and Force, T. (2015) The GSK-3 family as therapeutic target for myocardial diseases. Circ. Res. 116, 138–149 10.1161/CIRCRESAHA.116.303613 25552693 PMC4283216

[64] Avkiran, M., Cook, A.R. and Cuello, F. (2008) Targeting Na^+^/H^+^ exchanger regulation for cardiac protection: a RSKy approach? Curr. Opin. Pharmacol. 8, 133–140 10.1016/j.coph.2007.12.007 18222727

[65] Sweeney, M., Corden, B. and Cook, S.A. (2020) Targeting cardiac fibrosis in heart failure with preserved ejection fraction: mirage or miracle? EMBO Mol. Med. 12, e10865 10.15252/emmm.201910865 32955172 PMC7539225

