# Supplementary material for: The insulin receptor family and protein kinase B (Akt) are activated in the heart by alkaline pH and α1-adrenergic receptors
Source: Biochem J. 2021 Jun 8;478(11):2059–79. doi: 10.1042/BCJ20210144 (PMC8203208; doi:10.1042/BCJ20210144)

SUPPLEMENTAL TABLES AND FIGURES

Supplemental Table S1. Mouse body weights. p values assessed by repeated measures two-way ANOVA with Holm-Sidak post-test are given for body weights at time of mini-pump implantation (including the minipump) compared with the end of the study. Mice receiving linsitinib alone showed some weight gain over 4 d.

Study	Condition	Start weight (g)		End weight (g)		n	p
		Mean	SEM	Mean	SEM		
24 h study	Vehicle	26.88	0.74	26.40	0.81	6	0.491
(2 cohorts)	Linsitinib	25.78	1.03	25.97	1.01	6	0.622
	Phenylephrine	26.78	0.67	26.37	0.82	6	0.491
	Linsitinib/Phenylephrine	26.78	0.69	26.02	0.66	6	0.186
4 d study	Vehicle	25.44	0.63	25.71	0.49	7	0.347
(2 cohorts)	Linsitinib	25.86	0.78	26.33	0.84	7	**0.049**
	Phenylephrine	25.53	0.59	25.79	0.64	7	0.347
	Linsitinib/Phenylephrine	25.80	0.77	25.83	0.71	7	0.871

Supplemental Table S2. qPCR primers. Primers were from Invitrogen (supplied by Thermo Fisher Scientific).

Gene Symbol	Accession No.	Sense Primer (5'→3')	Antisense Primer (5'→3')
Col1a1	NM_007742	TCGTGGCTTCTCTGGTCTC	CCGTTGAGTCCGTCTTTGC
Col3a1	NM_009930.2	CTGGCACAAAAGGGACGAG	ACGTGGCCGAGAATTTCACC
Col4a1	NM_009931.2	TGTGGGCCAGCCAGGCATTG	CAGGGGGTCCGATCGCTCCA
Fn1	NM_010233	AAGAGGACGTTGCAGAGCTA	AGACACTGGAGACACTGACTAA
Myh7	NM_080728	CATGCCAACCGTATGGCTG	GTTCCACGATGGCGATGTTC
Nppa	NM_008725	GATGGATTTCAAGAACCTGCTAGA	CTTCCTCAGTCTGCTCACTCA
Nppb	NM_008726	TCCAGCAGAGACCTCAAAATTC	CAGTGCGTTACAGCCCAAA
Gapdh	NM_001289726.1	TCACCACCATGGAGAAGGC	GCTAAGCAGTTGGTGGTGCA

Supplemental Table S3. Antibodies used for immunoblotting and immunostaining. CST, Cell Signaling Technologies; SCBT, Santa Cruz Biotechnology Inc.; BD, BD Transduction Labs.

**Protein**	**Source**	**Cat. no.**	**Host**	**Dilution**
Phospho-PKB(T308)	CST	9275/4056	Rabbit	1/1000
Phospho-PKB(S473)	CST	4060	Rabbit	1/1000
Total PKB	CST	9272/4691	Rabbit	1/1000
Phospho-GSK3α(S21)/β(S9)	CST	9331	Rabbit	1/1000
Total GSK3β	CST	9332/9315	Rabbit	1/1000
Phospho-$p70^{S6K}$(T389)	CST	9234	Rabbit	1/1000
Total $p70^{S6K}$	SCBT	sc-230	Rabbit	1/1000
Phospho-Rps6(S235/S236)	CST	4858	Rabbit	1/1000
Total Rps6	CST	2217	Rabbit	1/1000
Phospho-JNKs	CST	4668	Rabbit	1/1000
Total JNKs	CST	9252	Rabbit	1/1000
Phospho-p38-MAPK	CST	8690	Rabbit	1/1000
Total p38-MAPK	CST	9212	Rabbit	1/1000
Phospho-ERK1/2	CST	4377	Rabbit	1/1000
Total ERK1/2	CST	4695	Rabbit	1/1000
Total INSRRα	Abnova	H00003645-M02	Mouse	1/750
Phospho-IGF-I Receptor β (Tyr1135/1136)/Insulin Receptor β (Tyr1150/1151)	CST	3024	Rabbit	1/1000
Phospho-p90RSK(S380)	CST	9335	Rabbit	1/1000
Total p90RSK1/RSK2/RSK3	CST	9355	Rabbit	1/1000
Flag	Sigma-Aldrich	F7425	Mouse	1/1000
Anti-Mouse immunoglobulins/HRP	Dako	P0260	Rabbit	1/5000
Anti-Rabbit immunoglobulins/HRP	Dako	P0448	Goat	1/5000
Troponin T	Invitrogen	MS-295-P1	Mouse	1/40
Anti-Mouse immunoglobulins/AlexaFluor 488	Invitrogen	A-11001	Goat	1/200

Supplemental Table S4. Echocardiography data for study of effects of linsitinib on the cardiac response to phenylephrine at 24 h. C57Bl/6J male mice were subjected to baseline echocardiography then implanted with minipumps to deliver vehicle (Control), linsitinib (2 mg/kg/d), phenylephrine (40 mg/kg/d) or linsitinib in the presence of phenylephrine. Echocardiograms were taken after 24 h. BPM, beats per minute; LV, left ventricular; AW, anterior wall, PW, posterior wall, IVS, interventricular septum; ID, internal diameter; WT, wall thickness (anterior wall + posterior wall).

Parameter	Unit	Vehicle		Linsitinib		PE		Linsitinib+PE	
		Mean	SD	Mean	SD	Mean	SD	Mean	SD
**Baseline**									
Heart Rate	BPM	511.4	41.1	513.4	46.1	504.6	45.4	514.1	25.4
Stroke Volume	µl	43.9	8.9	41.9	3.3	42.7	9.0	41.4	4.3
Ejection Fraction	%	57.0	2.7	62.3	4.6	54.7	2.6	59.4	1.9
Fractional Shortening	%	29.8	1.9	33.3	3.4	28.2	1.8	31.3	1.3
Cardiac Output	ml/min	22.6	5.8	21.6	3.5	21.4	3.9	21.4	2.3
LV Mass	mg	119	14	110.8	6.0	134.5	12.9	122.2	10.5
LVAW (d)	mm	0.827	0.075	0.837	0.041	0.879	0.053	0.858	0.046
LVAW (s)	mm	1.132	0.121	1.217	0.109	1.184	0.075	1.157	0.105
LVPW (d)	mm	0.714	0.061	0.726	0.033	0.800	0.039	0.794	0.054
LVPW (s)	mm	1.041	0.076	1.080	0.082	1.083	0.029	1.116	0.069
IVS (d)	mm	0.851	0.065	0.867	0.031	0.909	0.049	0.899	0.073
IVS (s)	mm	0.897	0.074	0.904	0.085	0.946	0.073	0.975	0.121
LVID (d)	mm	4.150	0.292	3.933	0.115	4.170	0.306	3.986	0.155
LVID (s)	mm	2.915	0.182	2.624	0.181	2.991	0.186	2.739	0.105
WT:ID (d)		0.373	0.045	0.394	0.024	0.405	0.040	0.416	0.028
WT:ID (s)		0.751	0.087	0.858	0.091	0.762	0.071	0.835	0.045
**24 h**									
Heart Rate	BPM	531.5	28.8	547.7	61.7	513.1	54.4	532.9	32.0
Stroke Volume	µl	45.7	7.3	41.3	3.9	40.6	10.8	38.2	4.0
Ejection Fraction	%	58.9	2.2	63.2	4.2	62.1	5.4	58.2	8.5
Fractional Shortening	%	31.0	1.5	33.8	3.2	33.1	3.8	30.5	5.8
Cardiac Output	ml/min	24.3	4.3	22.6	3.4	21.3	8.1	20.4	2.8
LV Mass	mg	124.8	16.9	109.5	16.5	124.7	18.8	108.2	12.8
LVAW (d)	mm	0.848	0.093	0.831	0.042	0.891	0.044	0.823	0.037
LVAW (s)	mm	1.200	0.111	1.131	0.080	1.176	0.097	1.085	0.050
LVPW (d)	mm	0.736	0.045	0.738	0.154	0.847	0.052	0.726	0.073
LVPW (s)	mm	1.047	0.070	1.117	0.115	1.182	0.075	1.059	0.047
IVS (d)	mm	0.835	0.082	0.868	0.034	0.933	0.053	0.888	0.090
IVS (s)	mm	0.939	0.064	0.949	0.074	1.022	0.079	1.022	0.077
LVID (d)	mm	4.171	0.247	3.881	0.067	3.861	0.386	3.900	0.104
LVID (s)	mm	2.880	0.162	2.566	0.112	2.582	0.281	2.715	0.285
WT:ID (d)		0.381	0.039	0.405	0.044	0.455	0.056	0.397	0.017
WT:ID (s)		0.782	0.072	0.879	0.087	0.924	0.131	0.798	0.098

Supplemental Table S5. Echocardiography data for study of effects of linsitinib on the cardiac response to phenylephrine at 4 d. C57Bl/6J male mice were subjected to baseline echocardiography then implanted with minipumps to deliver vehicle (Control), linsitinib (2 mg/kg/d), phenylephrine (40 mg/kg/d) or linsitinib in the presence of phenylephrine. Echocardiograms were taken after 4 d. BPM, beats per minute; LV, left ventricular; AW, anterior wall, PW, posterior wall, IVS, interventricular septum; ID, internal diameter; WT, wall thickness (anterior wall + posterior wall).

Parameter	Unit	Vehicle		Linsitinib		PE		Linsitinib+PE	
		Mean	SD	Mean	SD	Mean	SD	Mean	SD
**Baseline**									
Heart Rate	BPM	451.2	29.4	474.9	30.0	482.7	19.0	461.7	26.0
Stroke Volume	µL	32.0	3.0	37.6	15.5	34.9	3.8	34.3	3.7
Ejection Fraction	%	45.9	5.1	46.4	6.8	46.8	3.3	47.4	3.6
Fractional Shortening	%	22.7	3.2	23.1	4.4	23.2	1.9	23.5	2.1
Cardiac Output	ml/min	14.4	1.6	17.9	8.1	16.8	2.0	15.9	2.0
LV Mass	mg	104.3	13.6	112.4	24.3	116.4	16.3	110.2	9.3
LVAW (d)	mm	0.771	0.059	0.759	0.053	0.823	0.078	0.793	0.049
LVAW (s)	mm	1.056	0.083	1.058	0.076	1.098	0.087	1.072	0.058
LVPW (d)	mm	0.701	0.055	0.698	0.062	0.716	0.076	0.715	0.039
LVPW (s)	mm	0.945	0.065	0.944	0.149	0.976	0.074	0.965	0.050
IVS (d)	mm	0.791	0.037	0.817	0.039	0.811	0.062	0.841	0.034
IVS (s)	mm	0.879	0.045	0.849	0.059	0.870	0.064	0.924	0.054
LVID (d)	mm	3.979	0.173	4.165	0.422	4.092	0.152	4.042	0.178
LVID (s)	mm	3.074	0.215	3.183	0.161	3.140	0.139	3.087	0.187
WT:ID (d)		0.370	0.026	0.352	0.033	0.377	0.038	0.374	0.031
WT:ID (s)		0.659	0.094	0.629	0.048	0.663	0.057	0.663	0.054
**4 d**									
Heart Rate	BPM	484.9	31.2	488.5	46.4	483.2	36.6	470.9	30.9
Stroke Volume	µL	34.1	4.3	38.6	12.4	38.4	8.0	34.8	5.7
Ejection Fraction	%	49.3	4.0	49.5	8.8	61.8	7.9	61.7	4.4
Fractional Shortening	%	24.7	2.4	25.0	5.5	33.1	5.8	32.6	3.3
Cardiac Output	ml/min	16.5	2.8	18.9	6.6	18.5	4.3	16.4	3.2
LV Mass	mg	108.5	13.1	120.2	19.5	116.3	14.9	123.2	13.5
LVAW (d)	mm	0.807	0.062	0.814	0.069	0.900	0.077	0.928	0.051
LVAW (s)	mm	1.128	0.073	1.163	0.119	1.296	0.129	1.326	0.060
LVPW (d)	mm	0.701	0.031	0.729	0.110	0.800	0.069	0.913	0.184
LVPW (s)	mm	0.976	0.034	1.049	0.183	1.186	0.113	1.298	0.172
IVS (d)	mm	0.810	0.091	0.884	0.085	0.890	0.070	0.961	0.166
IVS (s)	mm	0.872	0.093	0.940	0.091	1.053	0.127	1.116	0.210
LVID (d)	mm	3.962	0.212	4.156	0.424	3.772	0.249	3.614	0.229
LVID (s)	mm	2.980	0.201	3.088	0.351	2.569	0.277	2.432	0.209
WT:ID (d)		0.381	0.021	0.375	0.058	0.453	0.056	0.513	0.075
WT:ID (s)		0.709	0.062	0.729	0.173	0.983	0.189	1.089	0.155

Supplemental Fig. S1. Linsitinib alone did not have any significant acute effects (24 h) on mouse hearts *in vivo*. C57Bl/6J male mice were subjected to baseline echocardiography then implanted with minipumps to deliver vehicle (Control) or linsitinib (2 mg/kg/d). Echocardiograms were taken after 24 h and mice were sacrificed. **A**, Linsitinib had no significant effect on phosphorylation of PKB/Akt or ERK1/2. Heart samples (25 µg) were immunoblotted for phosphorylated (Phospho-) or total kinases. **B**, Linsitinib had no significant effect on mRNA expression of hypertrophy (*Myh7, Nppa, Nppb*) or collagen (*Col1a1, Col3a1, Col4a1)* genes assessed by qPCR. **C**, Linsitinib had no effect on cardiac function or dimensions as determined using echocardiography. Quantitative analysis is shown for ejection fraction and fractional shortening plus systolic (s) and diastolic (d) cardiac dimensions at 24 h relative to baseline. LVPW, left ventricular posterior wall; LVID, LV internal diameter. Individual data points are shown with means ± SEM.

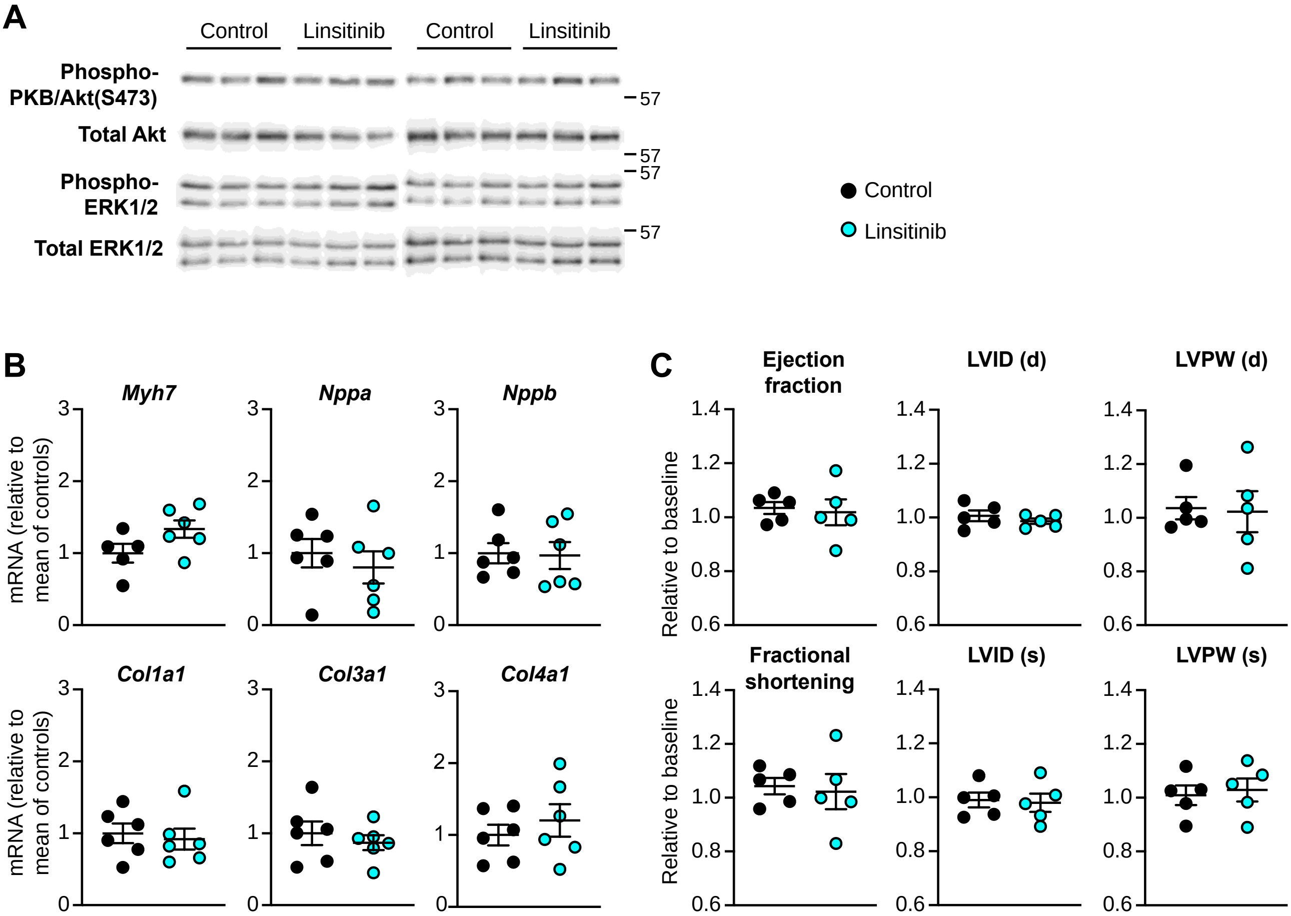

Supplemental Fig. S2. Linsitinib alone did not affect cardiac function/dimensions or mRNA expression at 4 d. C57Bl/6J male mice were treated with vehicle alone (Control) or linsitinib (2 mg/kg/d) using osmotic minipumps. **A-C**, Linsitinib did not affect cardiac function/dimensions assessed by echocardiography. Quantitative assessment of cardiac function (**A**) and left ventricular cardiac dimensions in diastole (**B**) and systole (**C**) at baseline (BL) and 4 d. WT:ID, ratio of wall thickness to internal diameter. Individual data points are shown with means ± SEM. **D**, Linsitinib did not affect mRNA expression of hypertrophy (*Myh7*, *Nppb*) or fibrosis-associated (*Col1a1*, *Col3a1*) genes. Individual data points are shown with means ± SEM.

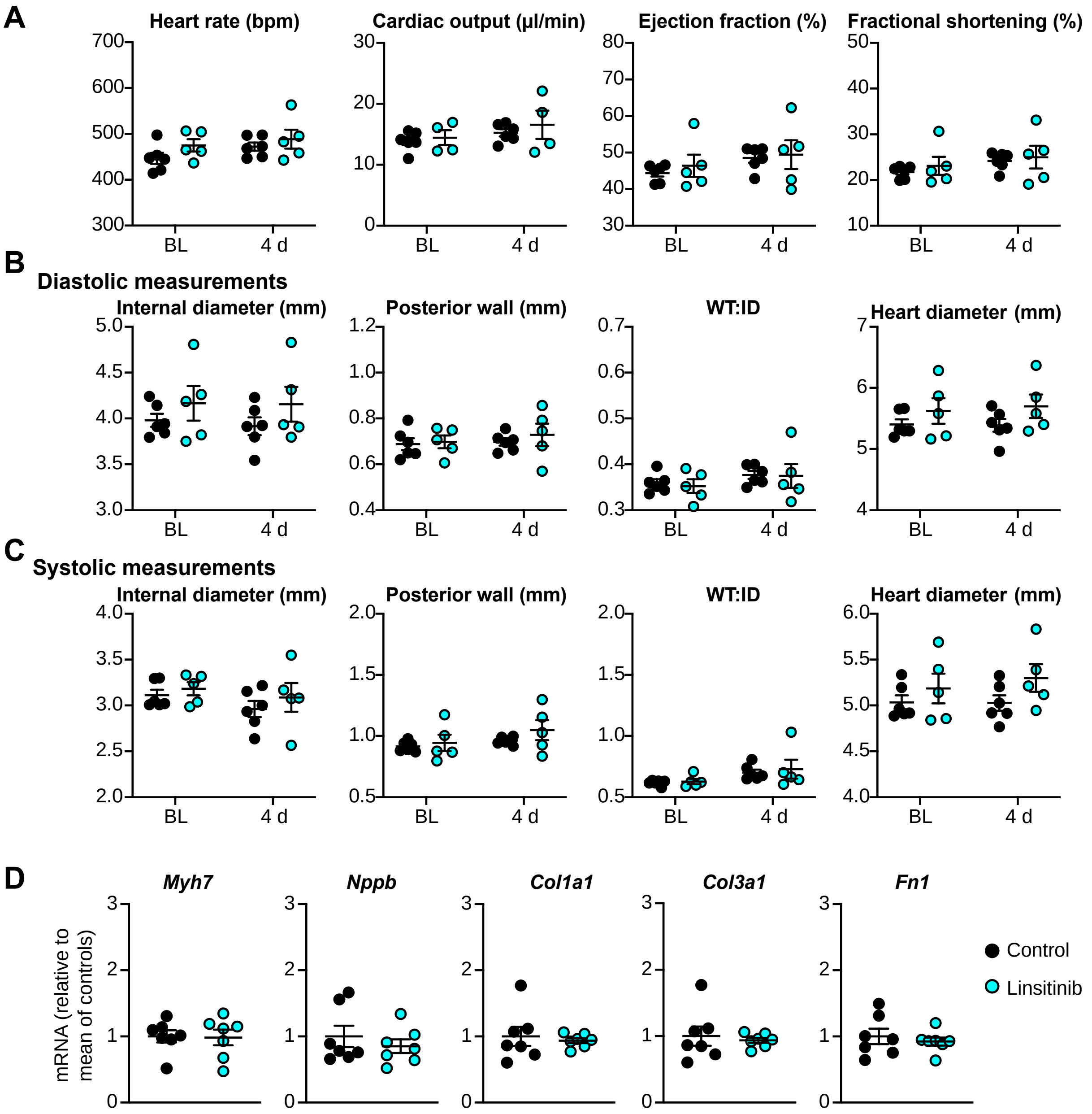

Supplement: Supplementary Tables S1-S5 and Figures S1-S2 [file BCJ-478-2059-s1.pdf]
